# Self-reported racial and ethnic discrimination in patient-provider interactions and the initiation of adjuvant therapies for breast cancer in the pathways study

**DOI:** 10.1007/s10549-026-08037-w

**Published:** 2026-08-10

**Authors:** Kevin R. Bitsie, Thomas A. Pearson, Marilyn L. Kwan, Lusine Yaghjyan, Lisa Scarton, Salma Shariff-Marco, Therese Y. Andraos, Lia L. D’Addario, Lawrence H. Kushi, Ting-Yuan David Cheng

**Affiliations:** 1 Dalla Lana School of Public Health, University of Toronto, 155 College St, 6th Floor, Toronto, ON M5T3M7 Canada; 2 Department of Epidemiology, University of Florida, Gainesville, FL USA; 3 Division of Research, Kaiser Permanente Northern California, Pleasanton, CA USA; 4 College of Nursing, University of Florida, Gainesville, FL USA; 5 Department of Epidemiology and Biostatistics, School of Medicine, University of California, San Francisco, San Francisco, CA USA; 6 Comprehensive Cancer Center, The Ohio State University, Columbus, OH USA; 7 Division of Cancer Prevention and Control, Department of Internal Medicine, The Ohio State University, 3650 Olentangy River Rd Suite 200, Columbus, OH 43214 USA

**Keywords:** Breast cancer, Discrimination, Race/ethnicity, Patient-provider, Breast cancer disparities, Adjuvant therapies, Chemotherapy, Radiation therapy, Hormonal therapy

## Abstract

**Purpose:**

Despite advancements in breast cancer adjuvant therapies, some patients with indications may not receive treatment. We examined the association of self-reported racial/ethnic discrimination in patient-provider interactions and the receipt of clinically indicated therapies.

**Methods:**

The Pathways Study is a prospective cohort of women diagnosed with invasive breast cancer from 2005 to 2013 at Kaiser Permanente Northern California. Racial/ethnic discrimination in patient-provider interactions was assessed from the Interpersonal Processes of Care survey at baseline, 6 months, and 24 months post-diagnosis. Logistic regression compared women who did not initiate clinically-indicated adjuvant therapy with those who did overall, and by race and ethnicity. Covariates included race and ethnicity, age at diagnosis, country of birth, education level, income, marital status, and American Joint Committee on Cancer staging.

**Results:**

Overall, 3,610 women had indication for hormonal therapy, 2,450 for chemotherapy, and 3,258 for radiation therapy. In multivariable analyses, women reporting discrimination were at increased odds of not initiating hormonal therapy (adjusted odds ratio [aOR] = 1.43, 95% CI = 1.05–1.93) than those who did not self-report discrimination, regardless of their race/ethnicity. An increased odds of non-initiation for reported discrimination was found for radiation therapy (aOR = 1.26, 95% CI = 0.99–1.61), although it was not statistically significant. There was no association for chemotherapy initiation (aOR = 0.96, 95% CI = 0.69–1.33).

**Conclusion:**

Self-reported racial/ethnic discrimination in patient-provider interactions was associated with the non-initiation of hormonal therapy in women with breast cancer. Further studies are needed to explore the impact of this association on breast cancer prognosis.

**Clinical trial registration:**

Not applicable.

**Supplementary Information:**

The online version contains supplementary material available at 10.1007/s10549-026-08037-w.

## Introduction

Despite advancements and benefits of adjuvant therapies for breast cancer survival [1–4], patients with therapy indications may not receive a treatment initially [5, 6] or experience discontinuation of treatment [3, 6, 7] Barriers associated with a lower likelihood of receipt of quality cancer treatment include inadequate health insurance coverage, low socioeconomic status, lack of a stable source of care, unreliable transportation, and health literacy challenges [8]. Racial and ethnic minorities are less likely to receive high-quality treatment consistent with clinical indication for specific types and stages of cancer [9–14]. Notably, racial or ethnic discrimination in healthcare settings, specifically from providers, may negatively impact healthcare-related outcomes [15], potentially impacting trust and decision-making that affect cancer outcomes [16]. Patients experiencing racism may seek to have less contact with the healthcare system, indirectly limiting their access to healthcare services [17]. A cross-sectional study among breast cancer survivors found that four of nine discrimination items (treated with less courtesy, treated with less respect, people act as if you are not smart, people act as if they think you are dishonest) were significantly associated with lower treatment receipt, based on univariate analyses [18].

The Pathways Study, a large prospective cohort of breast cancer survivors with detailed baseline and follow-up information on clinical and social measures, provided the opportunity to examine self-reported racial and ethnic discrimination in patient-provider interactions with the initiation of clinically-indicated chemotherapy, radiation therapy, and hormonal therapy, based on evidence-based guidelines. We hypothesized that participants reporting discrimination were less likely to initiate therapies than those who did not. Understanding barriers to adjuvant therapy initiation is essential for ensuring clinically indicated treatment, as treatment differences may result in racial or ethnic disparities in breast cancer morbidity and mortality [19–21].

## Methods

### Study population

Details of the Pathways Study have been previously described [22]. Briefly, the Pathways Study is a prospective cohort study of 4,504 women diagnosed with and treated for invasive breast cancer from late 2005 to 2013 in Kaiser Permanente Northern California (KPNC). Eligible participants were at least 21 years old at the time of their breast cancer diagnosis, had no prior cancer history (except non-melanoma skin cancer), and were KPNC members. Cancer diagnoses were ascertained through pathology reports and subsequently confirmed via medical record review [23]. A passive physician consent was obtained, allowing physicians one week to object to their patients being notified as potential participants. If no objections, the potential participant was assigned a field interviewer (14 total, with two Spanish-speaking and one Chinese-speaking) based within 65 miles of the interviewer’s home [23]. The Institutional Review Boards (IRB) at KPNC and the University of Florida approved this project reported here.

Participants completed an in-person baseline interview (on average two months post-diagnosis), mailed or telephone follow-up questionnaires at 6 and 24 months after baseline, with active follow-up ongoing. Baseline survey data included sociodemographic characteristics, health history, diet, physical activity, quality of life, and psychosocial factors. Sociodemographic and health history information collected included self-identified race and ethnicity, nativity, age, education, income, and marital status [23]. Clinical data, including breast cancer treatment data, were collected from Kaiser Permanente electronic health records and the KPNC Cancer Registry.

### Discrimination assessment

Among the 4,504 Pathways Study participants, only those with data on reported discrimination at baseline, 6-month, and 24-month follow-ups were included. The two questions asked were: “How often did doctors pay less attention to you because of your race or ethnicity?” and “How often did you feel discriminated against by doctors because of your race or ethnicity?” These questions are components of the Interpersonal Processes of Care (IPC) survey, an 18-item questionnaire assessing patient-provider interactions related to the quality of care over the past 12 months [23, 24]. IPC survey domains include: lack of clarity, elicited concerns, explained results, compassion, patient-centered decision-making, discrimination due to race and ethnicity, and disrespectful office staff [23, 24]. The IPC survey has been validated in patients from diverse racial and ethnic backgrounds, with the discrimination due to race and ethnicity domain showing an internal-consistency reliability of 0.77 [25]. Measures to ensure rigor and reproducibility of the agreement between the respective question responses have been previously described [22].

Responses to discrimination questions were recorded on a Likert scale ranging from 1 = ‘Never’ to 5 = ‘Always.’ Responses from baseline and two follow-ups were combined into “Ever” self-reported racial or ethnic discrimination in patient-provider interactions, defined as responses of “Rarely,” “Sometimes,” “Usually,” or “Always” at any time point, versus “Never”, defined as responses of “Never” at all three time points or at least once with missing responses for the other time points. We then combined these two questions to broaden reported discrimination. Participants with missing responses for both questions across all time points were excluded (*n* = 169), leaving an initial total of 4,335 participants. Further exclusions were made for missing or incompatible variables that determined clinically-indicated adjuvant therapy.

### Treatment indication and initiation

The National Comprehensive Cancer Network (NCCN) has established guidelines for clinicians to provide preventive, diagnostic, and supportive services in cancer care to improve patient outcomes [26]. Based on these guidelines, we constructed adjuvant therapy indications using clinical and pathological variables available from the KPNC Cancer Registry records: American Joint Committee on Cancer (AJCC) tumor staging, hormone receptor (HR) status, human epidermal growth factor receptor 2 (HER2) status, and type of surgery. Hormonal therapy was determined to be clinically indicated based on HR-positive tumors [27]. Chemotherapy was determined to be clinically indicated based on 2006 NCCN guidelines published during the Pathways Study recruitment period [26], along with a previous study [28], with criteria including age, tumor size, nodal status, estrogen receptor (ER), progesterone receptor (PR), and HER2 status (Supplemental Table 1). For radiation therapy, clinical indication was determined based on variables in the NCCN guidelines [26] and a previous study [29], with criteria including lumpectomy, post-mastectomy with AJCC stage III or higher, or post-mastectomy with any positive lymph nodes (Supplemental Table 2). The initiation of therapies (yes/no) was determined from electronic health records and the KPNC Cancer Registry [11, 27, 30]. The KPNC Cancer Registry follows standards of the NCI Surveillance, Epidemiology, and End Results (SEER) Program, which includes treatment with chemotherapy and radiation therapy. Neoadjuvant chemotherapy was not included. This information was augmented with additional data from electronic health records, including adjuvant therapy initiated after cases were reported to the SEER Program registries. Receipt of hormonal therapy was determined from medical oncologist documentation in the medical record.

### Statistical analysis

We performed descriptive analyses comparing the non-initiation and initiation of hormonal therapy, chemotherapy, and radiation therapy, examining demographic and clinical characteristics as frequencies and proportions for categorical variables, and means with standard deviations (SD) for continuous variables. We used univariable logistic regression to calculate odds ratios (OR) and 95% confidence intervals (CI) for the non-initiation and initiation of each adjuvant therapy group, comparing those with and without reported discrimination.

To assess independent associations in multivariable models, the associations of adjuvant therapy initiation differed by self-reported racial or ethnic discrimination in patient-provider interactions were calculated with adjusted ORs (aOR) and 95% CIs. Three models were performed for each adjuvant therapy to account for race and ethnicity differently. The first model examined the associations of non-initiation of adjuvant therapies by comparing ever versus never reported discrimination, adjusting for a two-category race and ethnicity variable (racial or ethnic minority women combined versus non-Hispanic White (NHW) women). The second model adjusted for a five-category race and ethnicity variable (the stratification by each racial or ethnic minority group of women versus NHW women). The third model analyzed subpopulations stratified by race and ethnicity.

Covariates for each model set included race and ethnicity (Hispanic, non-Hispanic Black, non-Hispanic Asian, American Indian, Alaska Native, Pacific Islander (AIANPI), and NHW women, who served as the reference group). AIANPI participants were combined due to small numbers (*n* = 97). Additionally, each model set for the adjuvant therapy group was adjusted for country of birth (US-born versus foreign-born), highest level of education completed (high school or less, some college, college graduate, post-graduate, and unknown), household income level (<$25K, $25K-$49K, $50K-$89K, ≥$90K, and unknown), marital status (married/lived as married, single/separated/divorced/widowed, and unknown), and AJCC staging (I, II, III, and IV). Chemotherapy models also adjusted for HR (positive/negative) and HER2 (positive/negative) status. For radiation therapy models, the type of surgery (lumpectomy/mastectomy) was adjusted. Age at diagnosis (years) was included as a continuous covariate. To assess the robustness of our results, we performed a sensitivity analysis based on SRREDPPI responses at baseline, at baseline plus the 6-month follow-up, and across all three timepoints. The varying timepoints were due to patients receiving care from different specialties during the treatment course and higher missing rates in follow-ups than at baseline (attention question: 5.8%, 38.0%, and 54.0% at baseline, 6 months, and 24 months, respectively; discriminated question: 5.4%, 38.0%, and 53.8% at baseline, 6 months, and 24 months, respectively). We also performed a sensitivity analysis excluding stage IV patients to account for different treatment courses from early-stage patients.

All statistical analyses were performed using RStudio 1.2 (RStudio Team, Boston, MA). Results were statistically significant at *p* < 0.05; tests were two-sided.

## Results

Supplemental Fig. 1 shows the flowchart of sample selection. Compared to the final sample for analysis, patients with missing SRREDPPI information (*n* = 169) were more likely to be from racial or ethnic minoritized groups, born outside the US, younger, high school or less educated, not reporting income, single/separated/widowed, and had more advanced disease (Supplemental Table 3). A similar pattern was observed for patients with missing criteria for hormonal therapy indication (Supplemental Table 3). Those missing criteria for chemotherapy and radiation therapy were more likely to have earlier-stage disease (Supplemental Tables 4 and 5).

### Chemotherapy initiation

Women who had their chemotherapy initiated were more likely to report discrimination than those who did not have their chemotherapy initiated, although the difference was not significant (14.9% vs. 12.6%, respectively; *p* = 0.13). Women who did not have their chemotherapy initiated were more likely to be NHW, US-born, older at diagnosis, earn $49K or less, have earlier-stage breast cancer, have HR-positivity, and HER2-negative breast cancer than their counterparts (Table 1). Women who ever reported discrimination were more likely to be racial or ethnic minorities, foreign-born, be younger, earn $49K or less, be single/separated/widowed, and have HR-negative breast cancer than those who never reported discrimination (Supplemental Table 6).

**Table 1:** Distribution of demographic variables as potential confounders stratified by chemotherapy initiation among breast cancer patients with an indication for chemotherapy (*n* = 2450)

Variable	Total (*n* = 2450)	Non-initiation of Chemotherapy (*n* = 788)	Initiation of Chemotherapy (*n* = 1662)	*P* for difference
	*n* (column %)	*n* (column %)	*n* (column %)	
SRREDPPI				0.13
Ever	346 (14.1)	99 (12.6)	247 (14.9)	
Never	2104 (85.9)	689 (87.4)	1415 (85.1)	
NHW vs. Minorities				< 0.0001
Non-Hispanic White	1498 (61.1)	531 (67.4)	967 (58.2)	
Racial or ethnic minorities	952 (38.9)	257 (32.6)	695 (41.8)	
Race and ethnicity strata				< 0.0001
Non-Hispanic White	1498 (61.1)	531 (67.4)	967 (58.2)	
Black	212 (8.7)	60 (7.6)	152 (9.1)	
Asian	381 (15.6)	103 (13.1)	278 (16.7)	
Hispanic	298 (12.2)	81 (10.3)	217 (13.1)	
American Indian, Alaska Native, or Pacific Islander	61 (2.5)	13 (1.6)	48 (2.9)	
Country of birth				0.016
US-Born	1879 (76.7)	628 (79.7)	1251 (75.3)	
Non-US Born	571 (23.3)	160 (20.3)	411 (24.7)	
Age at diagnosis				< 0.0001
Years, (mean ± SD)	54.4 ± 9.4	58.4 ± 7.9	52.6 ± 9.4	
Education level				0.31
High school or less	350 (14.3)	107 (13.6)	243 (14.6)	
Some college	836 (34.1)	304 (38.6)	532 (32.0)	
College graduate	710 (29.0)	198 (25.1)	512 (30.8)	
Post graduate	554 (22.6)	179 (22.7)	375 (22.6)	
Income level				0.006
< $25K	160 (6.5)	59 (7.5)	101 (6.1)	
$25K – $49K	415 (16.9)	152 (19.3)	263 (15.8)	
$50K – $89K	750 (30.6)	245 (31.1)	505 (30.4)	
≥ $90K	895 (36.5)	261 (33.1)	634 (38.1)	
Unknown	230 (9.4)	71 (9.0)	159 (9.6)	
Marital status				0.07
Married/lived as married	1604 (65.5)	496 (62.9)	1108 (66.7)	
Single/separated/widowed	843 (34.4)	291 (36.9)	552 (33.2)	
Unknown	3 (0.1)	1 (0.1)	2 (0.1)	
AJCC staging				< 0.0001
I	947 (38.7)	547 (69.4)	400 (24.1)	
II	1139 (46.5)	223 (28.3)	916 (55.1)	
III	327 (13.3)	12 (1.5)	315 (19.0)	
IV	37 (1.5)	6 (0.8)	31 (1.9)	
HR Status				< 0.0001
Positive	1997 (81.5)	757 (96.1)	1240 (74.6)	
Negative	453 (18.5)	31 (3.9)	422 (25.4)	
HER2 Status				< 0.0001
Positive	365 (14.9)	26 (3.3)	339 (20.4)	
Negative	2085 (85.1)	762 (96.7)	1323 (79.6)	

SRREDPPI = self-reported racial/ethnic discrimination in patient-provider interactions, NHW = Non-Hispanic White, SD = standard deviation, AJCC = American Joint Committee on Cancer, HR = hormone receptor, HER2 = human epidermal growth factor receptor 2

P for difference indicates descriptive univariate analyses comparing the non-initiation and initiation of chemotherapy examining demographics and clinical categorical variables

The multivariable model (Table 2) showed no association of reported discrimination with initiating chemotherapy overall (two-category race and ethnicity variable: aOR = 0.96, 95% CI = 0.69–1.33; five-category: aOR = 0.94, 95% CI = 0.67–1.32). Subpopulation analyses stratified by race and ethnicity groups found that reported discrimination was not associated with initiating chemotherapy.

**Table 2:** Results of univariable and multivariable logistic regression analyses for ever/never self-reported racial or ethnic discrimination in patient-provider interactions (SRREDPPI) and chemotherapy initiation by type of analysis and race and ethnicity-stratified subpopulations among breast cancer patients with an indication for chemotherapy in the Pathways Study (*n* = 2450)

Type of Analysis	SRREDPPI	Chemotherapy Initiation		Univariable Models	Multivariable Models^a^
		No	Yes	OR (95% CI)	aOR (95% CI)
		n (col %)	n (col %)		
Two-category race and ethnicity variable	Ever	99 (12.6)	247 (14.9)	0.82 (0.64–1.05)	0.96 (0.69–1.33)
	Never	689 (87.4)	1415 (85.1)		
Five-category race and ethnicity variable	Ever	99 (12.6)	247 (14.9)	0.82 (0.64–1.05)	0.94 (0.67–1.32)
	Never	689 (87.4)	1415 (85.1)		
Non-Hispanic White	Ever	35 (6.6)	56 (5.8)	1.15 (0.74–1.77)	1.24 (0.71–2.16)
	Never	496 (93.4)	911 (94.2)		
Racial or ethnic minority combined	Ever	64 (24.9)	191 (27.5)	0.88 (0.63–1.21)	0.79 (0.51–1.20)
	Never	193 (75.1)	504 (72.5)		
Black	Ever	21 (35.0)	50 (32.9)	1.10 (0.58–2.05)	0.98 (0.44–2.15)
	Never	39 (65.0)	102 (67.1)		
Asian	Ever	24 (23.3)	96 (34.5)	0.58 (0.34–0.96)	0.56 (0.28–1.09)
	Never	79 (76.7)	182 (65.5)		
Hispanic	Ever	18 (22.2)	38 (17.5)	1.35 (0.70–2.50)	1.05 (0.39–2.81)
	Never	63 (77.8)	179 (82.5)		
American Indian, Alaska Native, Pacific Islander	Ever	—	7 (14.6)	0.49 (0.02–3.15)	2.95 (0.04–143.77)
	Never	12 (92.3)	41 (85.4)		

^a^Adjusted for covariates including race or ethnicity (two-category race and ethnicity variable: Non-Hispanic White vs. racial or ethnic minority women combined; five-category race and ethnicity variable: race or ethnicity strata), country of birth, age, education level, income level, marital status, American Joint Committee on Cancer staging, hormone receptor (HR) status, and human epidermal growth factor receptor 2 (HER2) status

SRREDPPI = Self-reported racial/ethnic discrimination in patient-provider interactions, OR = odds ratio, aOR = adjusted odds ratio, col = column, Two-category race and ethnicity variable = examined the association of non-initiation of adjuvant therapies by comparing ever versus never reported discrimination, adjusting for combined racial or ethnic minority women versus Non-Hispanic White women. Five-category race and ethnicity variable = examined the association of non-initiation of adjuvant therapies by comparing ever versus never reported discrimination, adjusting the stratification by each racial/ethnic group of women with Non-Hispanic White women as the reference. P-interaction between SRREDPPI and race on non-initiation of chemotherapy = 0.22 (two-category race and ethnicity variable) and 0.37 (five-category race and ethnicity variable).To protect patient confidentiality and comply with institutional privacy policies, cell counts ≤ 5 are not reported and are indicated by ‘—’ in tables

### Radiation therapy initiation

Women who had no radiation therapy initiated were more likely to report ever discrimination than those who had radiation therapy initiated (15.0% vs. 11.8%, respectively, *p* = 0.01) (Table 3). Of the 3,258 women with a clinical indication for receiving radiation therapy, women who ever reported discrimination were more likely to be from a racially or ethnic minority group, be foreign-born, be younger, earn $49K or less, and be single/separated/widowed than those who never reported discrimination (Supplemental Table 7).

**Table 3:** Distribution of demographic variables as potential confounders stratified by radiation therapy initiation among breast cancer patients with an indication for radiation therapy (*n* = 3258)

Variable		Total (*n* = 3258)		Non-Initiation of Radiation Therapy (*n* = 1404)			Initiation of Radiation Therapy (*n* = 1854)		
		*n* (column %)		*n* (column %)			*n* (column %)		*P* for difference
SRREDPPI									0.006
Ever		429 (13.2)		211 (15.0)			218 (11.8)		
Never		2829 (86.8)		1193 (85.0)			1636 (88.2)		
NHW vs. Minorities									< 0.0001
Non-Hispanic White		2197 (67.4)		880 (62.7)			1317 (71.0)		
Racial or ethnic minorities		1061 (32.6)		524 (37.3)			537 (29.0)		
Race and ethnicity strata									0.0001
Non-Hispanic White		2197 (67.4)		880 (62.7)			1317 (71.0)		
Black		253 (7.8)		131 (9.3)			122 (6.6)		
Asian		375 (11.5)		194 (13.8)			181 (9.8)		
Hispanic		355 (10.9)		161 (11.5)			194 (10.5)		
American Indian, Alaska Native, and Pacific Islander		78 (2.4)		38 (2.7)			40 (2.2)		
Country of birth^a^									0.015
US-Born		2622 (80.6)		1102 (78.6)			1520 (82.0)		
Non-US Born		633 (19.4)		300 (21.4)			333 (18.0)		
Age at diagnosis^b^									< 0.0001
Years, (mean ± SD)		59.7 ± 11.8		58.1 ± 12.5			60.9 ± 11.1		
Education level									0.59
High school or less		506 (15.5)		236 (16.8)			270 (14.6)		
Some college		1120 (34.4)		455 (32.4)			665 (35.9)		
College graduate		882 (27.1)		397 (28.3)			485 (26.2)		
Post graduate		748 (23.0)		315 (22.4)			433 (23.4)		
Unknown		2 (0.1)		1 (0.1)			1 (0.1)		
Income level									0.92
< $25K		309 (9.5)		142 (10.1)			167 (9.0)		
$25K – $49K		622 (19.1)		265 (18.9)			357 (19.3)		
$50K – $89K		958 (29.4)		401 (28.6)			557 (30.0)		
≥ $90K		1024 (31.4)		442 (31.5)			582 (31.4)		
Unknown		345 (10.6)		154 (11.0)			191 (10.3)		
Marital status									0.73
Married/lived as married		2013 (61.8)		872 (62.1)			1141 (61.5)		
Single/separated/widowed		1242 (38.1)		531 (37.8)			711 (38.3)		
Unknown		3 (0.01)		1 (0.1)			2 (0.1)		
AJCC staging									< 0.0001
I		1682 (51.6)		373 (26.6)			1309 (70.6)		
II		1156 (35.5)		721 (51.4)			435 (23.5)		
III		387 (11.9)		290 (20.7)			97 (5.2)		
IV		33 (1.0)		20 (1.4)			13 (0.7)		
Surgery									< 0.0001
Lumpectomy		2561 (78.6)		833 (59.3)			1728 (93.2)		
Mastectomy		697 (21.4)		571 (40.7)			126 (6.8)		

^a^*n* = 3 women with missing country of birth. ^b^*n* = 2 women with missing age at diagnosis. SRREDPPI = self-reported racial/ethnic discrimination in patient-provider interactions, NHW = Non-Hispanic White, SD = standard deviation, AJCC = American Joint Committee on Cancer. P for difference indicates descriptive univariate analyses comparing the non-initiation and initiation of radiation therapy examining demographics and clinical categorical variables

In univariate analysis, reported discrimination was associated with the non-initiation of radiation therapy (OR = 1.33, 95% CI = 1.08–1.63) (Table 4). However, after adjustment of covariates, the association was not significant (aOR = 1.26, 95% CI = 0.99–1.61). The null association was also observed among race and ethnicity-stratified analyses (NHW: aOR = 1.17, 95% CI = 0.79–1.72; combined racial or ethnic minority women: aOR = 1.34, 95% CI = 0.97–1.84; Black: aOR = 0.88, 95% CI = 0.48–1.59; Asian: aOR = 1.43, 95% CI = 0.83–2.48; Hispanic: aOR = 1.70, 95% CI = 0.90–3.26; AIANPI: aOR = 3.41, 95% CI = 0.41–50.94).

**Table 4:** Results of univariable and multivariable logistic regression analyses for ever/never self-reported racial or ethnic discrimination in patient-provider interactions (SRREDPPI) and radiation therapy initiation by type of analysis and race and ethnicity-stratified subpopulations among breast cancer patients with an indication for radiation therapy in the Pathways Study (n = 3258)

Type of Analysis	SRREDPPI	Radiation Therapy Initiation		Univariable Models	Multivariable Models^a^
		No	Yes	OR (95% CI)	aOR (95% CI)
		n (col %)	n (col %)		
Two-category race and ethnicity variable	Ever	211 (15.0)	218 (11.8)	1.33 (1.08 – 1.63)	1.26 (0.99 – 1.61)
	Never	1193 (85.0)	1636 (88.2)		
Five-category race and ethnicity variable	Ever	211 (15.0)	218 (11.8)	1.33 (1.08 – 1.63)	1.22 (0.95 – 1.56)
	Never	1193 (85.0)	1636 (88.2)		
Non-Hispanic White	Ever	59 (6.7)	86 (6.5)	1.03 (0.73 – 1.45)	1.17 (0.79 – 1.72)
	Never	821 (93.3)	1231 (93.5)		
Racial or ethnic minority combined	Ever	152 (29.0)	132 (24.6)	1.25 (0.96 – 1.65)	1.34 (0.97 – 1.84)
	Never	372 (71.0)	405 (75.4)		
Black	Ever	45 (34.4)	46 (37.7)	0.86 (0.52 – 1.45)	0.88 (0.48 – 1.59)
	Never	86 (65.6)	76 (62.3)		
Asian	Ever	67 (34.5)	51 (28.2)	1.34 (0.87 – 2.09)	1.43 (0.83 – 2.48)
	Never	127 (65.5)	130 (71.8)		
Hispanic	Ever	33 (20.5)	32 (16.5)	1.31 (0.76 – 2.24)	1.70 (0.90 – 3.26)
	Never	128 (79.5)	162 (83.5)		
American Indian, Alaska Native, and Pacific Islander	Ever	7 (18.4)	—	2.78 (0.71 – 13.77)	3.41 (0.41 – 50.94)
	Never	31 (81.6)	37 (92.5)		

^a^Adjusted for covariates including race or ethnicity (two-category race and ethnicity variable: Non-Hispanic White vs. racial or ethnic minority women combined; five-category race and ethnicity variable: race or ethnicity strata), country of birth, age at diagnosis, education level, income level, marital status, American Joint Committee on Cancer staging and type of surgery

SRREDPPI = self-reported racial/ethnic discrimination in patient-provider interactions, OR = odds ratio, aOR = adjusted odds ratio, col = column, Two-category race and ethnicity variable = examined the association of non-initiation of adjuvant therapies by comparing ever versus never reported discrimination, adjusting for combined racial or ethnic minority women versus NHW women. Five-category race and ethnicity variable = examined the association of non-initiation of adjuvant therapies by comparing ever versus never reported discrimination, adjusting the stratification by each racial/ethnic group of women with NHW women as the reference. P-interaction between SRREDPPI and race on non-initiation of radiation therapy = 0.59 (two-category race and ethnicity variable) and 0.42 (five-category race and ethnicity variable) To protect patient confidentiality and comply with institutional privacy policies, cell counts ≤5 are not reported and are indicated by '—' in tables

### Hormonal therapy initiation

Women who had no hormonal therapy initiated were more likely to report ever discrimination than those who had hormonal therapy initiated (16.3% vs. 12.3%, respectively, *p* = 0.02) (Table 5). Of the 3,610 women with a clinical indication for receiving hormonal therapy, those who reported discrimination were more likely to be from racial or ethnic minorities, be foreign-born, younger, earn $49K or less, and be single/separated/widowed than those who never reported discrimination (Supplemental Table 8).

**Table 5:** Distribution of demographic variables as potential confounders stratified by hormonal therapy initiation among HR-positive breast cancer patients (*n* = 3610)

Variable	Total (*n* = 3610)	Non-Initiation of Hormonal Therapy (*n* = 404)	Initiation of Hormonal Therapy (*n* = 3206)	
	*n* (column %)	*n* (column %)	*n* (column %)	*P* for difference
SRREDPPI				0.023
Ever	461 (12.8)	66 (16.3)	395 (12.3)	
Never	3149 (87.2)	338 (83.7)	2811 (87.7)	
NHW vs. Minorities				0.46
Non-Hispanic White	2444 (67.7)	280 (69.3)	2164 (67.5)	
Racial or ethnic minorities	1166 (32.3)	124 (30.7)	1042 (32.5)	
Race and ethnicity strata				0.91
Non-Hispanic White	2444 (67.7)	280 (69.3)	2164 (67.5)	
Black	215 (6.0)	21 (5.2)	194 (6.1)	
Asian	475 (13.2)	43 (10.6)	432 (13.5)	
Hispanic	402 (11.1)	46 (11.4)	356 (11.1)	
American Indian, Alaska Native, and Pacific Islander	74 (2.0)	14 (3.5)	60 (1.9)	
Country of birth^a^				0.58
US-Born	2861 (79.3)	324 (80.4)	2537 (79.2)	
Non-US Born	745 (20.7)	79 (19.6)	666 (20.8)	
Age at diagnosis^b^				< 0.0001
Years, (mean ± SD)	59.8 ± 12.0	63.9 ± 13.6	59.3 ± 11.7	
Education level				0.018
High school or less	557 (15.4)	71 (17.6)	486 (15.2)	
Some college	1211 (33.5)	149 (36.9)	1062 (33.1)	
College graduate	1010 (28.0)	105 (26.0)	905 (28.2)	
Post graduate	829 (23.0)	79 (19.6)	750 (23.4)	
Unknown	3 (0.1)	0 (0)	3 (0.1)	
Income level				0.027
< $25K	337 (9.3)	57 (14.1)	280 (8.7)	
$25K – $49K	685 (19.0)	85 (21.0)	600 (18.7)	
$50K – $89K	1045 (28.9)	105 (26.0)	940 (29.3)	
≥ $90K	1158 (32.1)	99 (24.5)	1059 (33.0)	
Unknown	385 (10.7)	58 (14.4)	327 (10.2)	
Marital status				0.018
Married/lived as married	2228 (61.7)	226 (55.9)	2002 (62.4)	
Single/separated/widowed	1371 (38.0)	178 (44.1)	1193 (37.2)	
Unknown	11 (0.3)	0 (0)	11 (0.3)	
AJCC staging				< 0.0001
I	2028 (56.2)	302 (74.8)	1726 (53.8)	
II	1199 (33.2)	77 (19.1)	1122 (35.0)	
III	330 (9.1)	21 (5.2)	309 (9.6)	
IV	53 (1.5)	4 (1.0)	49 (1.5)	

^a^ *n* = 4 women with missing country of origin. ^b^ *n* = 3 women with missing age at diagnosis. SRREDPPI = Self-reported racial/ethnic discrimination in patient-provider interactions. NHW = Non-Hispanic White. SD = standard deviation. AJCC = American Joint Committee on Cancer. P for difference indicates descriptive univariate analyses comparing the non-initiation and initiation of hormonal therapy examining demographics and clinical categorical variables

Overall, women who reported discrimination had significantly higher odds of not initiating hormonal therapy than those who never reported discrimination (aOR = 1.43, 95% CI = 1.05–1.93) (Table 6). Subpopulation race and ethnicity-stratified analyses found no significant association except among AIANPI women (aOR = 20.87, 95% CI = 1.66-414.47).

**Table 6:** Results of univariable and multivariable logistic regression analyses for ever/never self-reported racial or ethnic discrimination in patient-provider interactions (SRREDPPI) and hormonal therapy initiation by type of analysis and race and ethnicity-stratified subpopulations among HR-positive breast cancer patients in the Pathways Study (*n* = 3610)

Type of Analysis	SRREDPPI	Hormonal Therapy Initiation		Univariable Models	Multivariable Models^a^
		No	Yes	OR (95% CI)	aOR (95% CI)
		n (col %)	n (col %)		
Two-category race and ethnicity variable	Ever	66 (16.3)	395 (12.3)	1.39 (1.04–1.83)	1.43 (1.05–1.93)
	Never	338 (83.7)	2811 (87.7)		
Five-category race and ethnicity variable	Ever	66 (16.3)	395 (12.3)	1.39 (1.04–1.83)	1.50 (1.10–2.02)
	Never	338 (83.7)	2811 (87.7)		
Non-Hispanic White	Ever	28 (10.0)	145 (6.7)	1.55 (0.99–2.33)	1.44 (0.91–2.21)
	Never	252 (90.0)	2019 (93.3)		
Racial or ethnic minority combined	Ever	38 (30.6)	250 (24.0)	1.40 (0.92–2.09)	1.35 (0.88–2.05)
	Never	86 (69.4)	792 (76.0)		
Black	Ever	10 (47.6)	64 (33.0)	1.85 (0.73–4.60)	1.47 (0.55–3.90)
	Never	11 (52.4)	130 (67.0)		
Asian	Ever	15 (34.9)	126 (29.2)	1.30 (0.66–2.49)	1.27 (0.61–2.52)
	Never	28 (65.1)	306 (70.8)		
Hispanic	Ever	10 (21.7)	55 (15.4)	1.52 (0.68–3.14)	1.63 (0.70–3.51)
	Never	36 (78.3)	301 (84.6)		
American Indian, Alaska Native, Pacific Islander	Ever	—	—	3.00 (0.55–14.19)	20.87 (1.66–414.47)
	Never	11 (78.6)	55 (91.7)		

^a^Adjusted for covariates including race or ethnicity (primary: NHW vs. WOC; sub-analysis: race or ethnicity strata), country of birth, age, education level, income level, marital status, and AJCC staging

SRREDPPI = Self-reported racial/ethnic discrimination in patient-provider interactions. OR = odds ratio. aOR = adjusted odds ratio. Two-category race and ethnicity variable = examined the association of non-initiation of hormonal therapy by comparing ever versus never reported discrimination, adjusting for covariates including combined racial or ethnic minority women versus Non-Hispanic White women. Five-category race and ethnicity variable = examined the association of non-initiation of hormonal therapy by comparing ever versus never reported discrimination, adjusted the stratification by each racial/ethnic group of women with Non-Hispanic White women as the reference. P-interaction between SRREDPPI and race on non-initiation of hormonal therapy = 0.96 (two-category race and ethnicity variable) and 0.77 (five-category race and ethnicity variable). To protect patient confidentiality and comply with institutional privacy policies, cell counts ≤ 5 are not reported and are indicated by ‘—’ in tables

Our sensitivity analysis showed similar aORs for ever vs. never discrimination and treatment non-initiation based on participants’ SRREDPPI responses at baseline only and at baseline plus 6-month follow-up across all three treatment initiations (Supplemental Tables 9–14). Results were similar before and after removing patients with stage IV disease (Supplemental Tables 15–17).

## Discussion

In a racially and ethnically diverse cohort of breast cancer survivors, we found that self-reported racial or ethnic discrimination in patient-provider interactions was significantly associated with the non-initiation of hormonal therapy but not with chemotherapy and radiation therapy.

Several studies support our finding that NHW women were less likely to initiate chemotherapy than racial or ethnic minorities. A recent study reported Hispanic White women and Black women with triple-negative cancer were more likely to initiate chemotherapy than NHW women, after adjusting for patient and tumor characteristics [31]. Another study noted NHW women had lower chemotherapy initiation rates than Black women in Washington, DC, and Detroit, MI [32]. Notably, among those with clinical indications for chemotherapy and more chemotherapy communication with providers (versus less), Black women were more likely to initiate chemotherapy than White women [32].

Differences in higher non-initiation of chemotherapy in NHW women compared to other groups of women may reflect financial strain [12, 33]. In our study, although NHW women, as well as Asian women, had higher income levels than the other race and ethnicity groups, income was not a determinant of chemotherapy initiation in multivariable models. This finding may be unique to our study population, as all participants were Kaiser Permanente members and had access to cancer treatment. Factors beyond income, such as transportation, family size, and other living expenses, are determinants of financial burden; however, we were unable to include these factors in our analysis.

The reason remains unclear that among combined minority women and Asian women, reporting discrimination was associated with a higher rate of chemotherapy initiation. Notably, Asian women with breast cancer represent a heterogeneous group with respect to ethnicity and nativity [34]. In our sample, 74% of Asian women were born outside the US. However, we observed no difference in chemotherapy initiation based on country of birth in multivariable models. Cultural differences between Asian women and their providers might explain this [34, 35]. alongside factors such as acculturation and patient-provider communication, which warrant further research.

Additionally, we found increased odds of non-initiation of radiation therapy among patients who reported discrimination overall and when stratified by race and ethnicity, though not significant. Besides the smaller numbers of AIANPI women, Hispanic women had the highest odds of non-initiation of radiation therapy of all therapies across all subpopulations. Previous studies also showed that Hispanic women are less likely to initiate radiation therapy than NHW women [14, 36, 37], with one noting variations in treatment by country of origin.^38^ Our study found no difference in country of birth in multivariable models. Another study using SEER-Medicare data found Hispanic women living far from radiation therapy providers had the lowest initiation rates at 2, 6, and 24 months [39]. To our knowledge, there was no literature on discrimination and initiation of adjuvant radiation therapy in patients with breast cancer. While our study did not examine residential location or census tract characteristics to identify potential place-based barriers (e.g., distance, transportation) to therapy initiation, it underscores the multifactorial and complex impact that racism may have on breast cancer morbidity and mortality.

Our findings on reported discrimination and non-initiation of hormonal therapy were inconsistent with a previous study from Sheppard et al. which found that overall perceived discrimination was not associated with initiation of endocrine therapy, despite Black women with breast cancer reporting more experiences with discrimination than White women with breast cancer [40]. Additionally, this study found that patients reporting higher levels of communication with their provider were more likely to initiate endocrine therapy than those reporting less communication, with no racial difference. A previous study showed that Black women rated the quality of provider communication lower than their White counterparts [18]. Conversely, Latinas and Black women had higher odds of initiating hormonal therapy than NHW women in the Detroit and Los Angeles SEER registries from 2005 to 2007 who were influenced by recurrence concerns and adequate education on potential side effects [6]. The present study found that reported discrimination was significantly associated with the non-initiation of hormonal therapy in all women, but the associations were similar and nonsignificant when stratified by race and ethnicity, suggesting that the quality of patient-provider communication may influence hormonal therapy initiation, and race and ethnicity may play a lesser role. Given these equivocal findings from our study and the Sheppard et al. study, which differ in discrimination measurement scales (Race-Based Experiences scale) and the proportion of non-White participants to White participants, further research is needed to elucidate the nuances of discrimination and initiation of hormonal therapy to examine whether patients may be opting out of suggested treatments, distrust in providers/healthcare system, or not be offered life-saving drugs. Ultimately, these studies indicate that addressing patients’ informational needs during provider interactions may improve treatment and outcomes.

We observed an association with hormonal therapy, but not with chemotherapy or radiation therapy, among all patients. A reason for this between-treatment difference may be that chemotherapy and radiation therapy have objective clinical criteria, such as Oncotype DX for chemotherapy, which can lead to more objective decision-making by clinicians when initiating treatment. However, data on use of Oncotype Dx were only available for a small subset (12%) and not suitable for further analyses.

Our study has several strengths. The KPNC cancer registry and electronic health records provide extensive clinical variables and tumor characteristics to determine therapy indications based on NCCN guidelines. These guidelines offer evidence-based recommendations for clinicians, patients, and others involved in cancer management and treatment decision-making. Additionally, the inclusion of reported discrimination across three cross-sectional time points allowed evaluation of discrimination experienced without repeated reports at each time point.

This study also has limitations. We did not differentiate between primary, adjuvant, or combined treatment. A previous study classified treatments as breast-conserving surgery or mastectomy and radiation therapy as primary, while chemotherapy or hormonal therapy alone as adjuvant, and also used combined treatment, i.e., chemotherapy and hormonal therapy [41]. Future studies encompassing comprehensive treatments, whether primary or adjuvant, may reveal other disparities, providing further insights into patient-provider interactions. Despite significant associations for the hormone therapy analysis among AIANPI women, results should be interpreted with caution due to small sample sizes, as racial or ethnic minority women were not proportional to NHW women in the given geographic group. Additionally, the timing of discrimination assessment relative to the start of treatment may have been a limitation, where a participant reported discrimination within the past 12 months at the 24-month follow-up when reported discrimination may have been assessed after initiation of adjuvant therapies. Despite this, the proportion of women who reported discrimination at the 24-month follow-up was considerably less than at baseline (attention question: baseline 9.3%, 6-month 3.2%, 24-month 2.6%; discrimination question: baseline 6.0%, 6-month 2.2%, 24-month 1.7%). Sensitivity analysis showed most associations were based on baseline responses. When comparing final analytic and excluded samples, for each adjuvant therapy, race/ethnicity and AJCC staging differ between the analytic sample and the sample excluded based on NCCN criteria. For hormonal therapy, a higher percentage of Black women and advanced stage cases were excluded than in the final analytic sample (Supplemental Table 3). A higher percentage of Asian American women and early stage cases were in the excluded sample than in the final radiation therapy sample (Supplemental Table 4). Also, a higher percentage of NHW, women with lower income, and an earlier stage were in the excluded sample than the chemotherapy analytic sample (Supplemental Table 5). Given this, the findings cannot be generalized to the broader population or those not meeting NCCN criteria. Additionally, we did not explore patients’ perspectives on therapy decision-making – whether provider-driven, patient-driven, or shared. Women diagnosed with invasive breast cancer who made provider-driven decisions had significantly lower odds of reporting excellent or best health care than those with shared decision-making [42]. Notably, a small proportion of patients–1.5%, 1.0%, and 1.5%–had stage IV disease in chemotherapy, radiation therapy, and hormone therapy analyses, respectively, and may receive these treatments without surgery. We performed sensitivity analyses by excluding patients with stage IV disease and found no difference in estimates. We were unable to account for transportation or other access barriers at treatment sites. Additionally, the generalizability of our study to current chemotherapy practices and decision-making may be limited, as indicated by the 2006 NCCN guidelines determining clinical indication for chemotherapy, and the patient cohort was diagnosed, recruited, and surveyed between 2005 and 2013. Notably, frailty and performance that could affect treatment initiation were not collected in the Pathways Study. Data on use of Oncotype DX were only available for a small subset (12%) and not suitable for further analyses. Finally, although we specified a priori hypotheses, given the number of analyses, we cannot rule out the possibility of chance findings, particularly in the race and ethnicity-stratified analyses.

In this prospective cohort study of women with breast cancer, self-reported discrimination was significantly associated with the non-initiation of hormonal therapy. These findings suggest providers must ensure the elimination of any perceived discrimination by patients to build trust, improve patient-provider relationships, and facilitate optimal care delivery. Future studies should further investigate how self-reported racial or ethnic discrimination in patient-provider interactions affects breast cancer outcomes.

## Supplementary Information

Below is the link to the electronic supplementary material.

Supplementary Material 1

## Data Availability

The datasets generated during and/or analyzed during the current study are not publicly available due to patient confidentiality but are available from the Pathways Study authors upon reasonable request.
