# Supplementary material for: Self-reported racial and ethnic discrimination in patient-provider interactions and the initiation of adjuvant therapies for breast cancer in the pathways study
Source: Breast Cancer Res Treat. 2026 Aug 10;218(3):36. doi: 10.1007/s10549-026-08037-w (PMC13457354; doi:10.1007/s10549-026-08037-w)
Supplement: Supplementary file 1 [file 10549_2026_8037_MOESM1_ESM.docx]

Table of Contents for Self-Reported Racial and Ethnic Discrimination in Patient-Provider Interactions and the Initiation of Adjuvant Therapies for Breast Cancer in the Pathways Study

Kevin R. Bitsie, MPH, PhD, Thomas A. Pearson, MD, MPH, PhD, Marilyn Kwan, PhD, Lusine Yaghjyan, MD, MPH, PhD, Lisa Scarton, PhD, RN, Salma Shariff-Marco, PhD, MPH, Therese Y. Andraos, MD, Lia L. D’Addario, MPH, Lawrence H. Kushi, ScD, Ting-Yuan David Cheng, PhD, MHS, MS

Breast Cancer Research and Treatment

March 2026

Table of Contents

Supplemental Table 1. Clinical indications of chemotherapy based on National Comprehensive Cancer Network guidelines among breast cancer patients that responded to discrimination due to race or ethnicity domain questions in the Pathways Study (n = 4335)……………………………5

Supplemental Table 2. Clinical indications for radiation therapy based on National Comprehensive Cancer Network guidelines among breast cancer patients that responded to discrimination due to race or ethnicity domain questions in the Pathways Study (n = 4335)…….7

Supplemental Table 3. Comparing final vs. excluded samples for hormonal therapy (n = 4504)..9

Supplemental Table 4. Comparing final vs. excluded samples for radiation therapy (n = 4504).10

Supplemental Table 5. Comparing final vs. excluded samples for chemotherapy (n = 4504)…..12

Supplemental Table 6. Distribution of demographic variables stratified by ever vs. never self-reported racial or ethnic discrimination in patient-provider interactions among chemotherapy indicated breast cancer patients (n = 2450)……………………………………………………...14

Supplemental Table 7. Distribution of demographic variables stratified by ever vs. never self-reported racial or ethnic discrimination in patient-provider interactions among radiation therapy indicated breast cancer patients (n = 3258)……………………………………………………...15

Supplemental Table 8. Distribution of demographic variables stratified by ever vs. never self-reported racial or ethnic discrimination in patient-provider interactions among HR-positive breast cancer patients (n = 3,610)……………………………………………………………………….16

Supplemental Table 9. Results of univariable and multivariable logistic regression analyses for ever/never self-reports of racial or ethnic discrimination in patient-provider interactions (SRREDPPI) and hormonal therapy initiation by type of analysis and race and ethnicity-stratified subpopulations among HR-positive breast cancer patients in the Pathways Study (n = 3,554) **(Baseline only**)…………………………………………………………………………………..17

Supplemental Table 10. Results of univariable and multivariable logistic regression analyses for ever/never self-reports of racial or ethnic discrimination in patient-provider interactions (SRREDPPI) and hormonal therapy initiation by type of analysis and race and ethnicity-stratified subpopulations among HR-positive breast cancer patients in the Pathways Study (n = 3,602) **(Baseline and 6-month follow-up)**……………………………………………………………..18

Supplemental Table 11. Results of univariable and multivariable logistic regression analyses for ever/never self-reported racial or ethnic discrimination in patient-provider interactions (SRREDPPI) and radiation therapy initiation by type of analysis and race and ethnicity-stratified subpopulations among radiation therapy indicated breast cancer patients in the Pathways Study (n = 3,202) **(Baseline only)**……………………………………………………………………...19

Supplemental Table 12. Results of univariable and multivariable logistic regression analyses for ever/never self-reported racial or ethnic discrimination in patient-provider interactions (SRREDPPI) and radiation therapy initiation by type of analysis and race and ethnicity-stratified subpopulations among radiation therapy indicated breast cancer patients in the Pathways Study (n = 3,247) **(Baseline and 6-month)**……………………………………………………………20

Supplemental Table 13. Results of univariable and multivariable logistic regression analyses for ever/never self-reported racial or ethnic discrimination in patient-provider interactions (SRREDPPI) and chemotherapy initiation by type of analysis and race and ethnicity-stratified subpopulations among chemotherapy indicated breast cancer patients in the Pathways Study (n = 2412) **(Baseline only)**……………………………………………………………………………21

Supplemental Table 14. Results of univariable and multivariable logistic regression analyses for ever/never self-reported racial or ethnic discrimination in patient-provider interactions (SRREDPPI) and chemotherapy initiation by type of analysis and race and ethnicity-stratified subpopulations among chemotherapy indicated breast cancer patients in the Pathways Study (n = 2441) **(Baseline and 6-month)**………………………………………………………………….22

Supplemental Table 15. Results of univariable and multivariable logistic regression analyses for ever/never self-reports of racial or ethnic discrimination in patient-provider interactions (SRREDPPI) and hormonal therapy initiation by type of analysis and race and ethnicity-stratified subpopulations among HR-positive breast cancer patients in the Pathways Study (n = 3,557) **(removal of stage IV)**…………………………………………………………………………...23

Supplemental Table 16. Results of univariable and multivariable logistic regression analyses for ever/never self-reported racial or ethnic discrimination in patient-provider interactions (SRREDPPI) and radiation therapy initiation by type of analysis and race and ethnicity-stratified subpopulations among radiation therapy indicated breast cancer patients in the Pathways Study (n = 3,225) **(removal of stage IV)**………………………………………………………………24

Supplemental Table 17. Results of univariable and multivariable logistic regression analyses for ever/never self-reported racial or ethnic discrimination in patient-provider interactions (SRREDPPI) and chemotherapy initiation by type of analysis and race and ethnicity-stratified subpopulations among chemotherapy indicated breast cancer patients in the Pathways Study (n = 2413) **(removal of stage IV)**…………………………………………………………………….25

Supplemental Table 18. Results of multivariable logistic regression analyses (with and without adjusting for Charlson Comorbidity Index) for ever/never self-reports of racial or ethnic discrimination in patient-provider interactions (SRREDPPI) and hormonal therapy initiation by type of analysis and race and ethnicity-stratified subpopulations among HR-positive breast cancer patients in the Pathways Study (n = 3610)……………………………………………….26

Supplemental Table 19. Results of multivariable logistic regression analyses (with and without adjusting for Charlson Comorbidity Index) for ever/never self-reported racial or ethnic discrimination in patient-provider interactions (SRREDPPI) and chemotherapy initiation by type of analysis and race and ethnicity-stratified subpopulations among chemotherapy indicated breast cancer patients in the Pathways Study (n = 2450)……………………………………………….27

Supplemental Figure 1. Inclusion criteria for the analysis of self-reported racial/ethnic discrimination in patient-provider interactions in the Pathways Study, for participants with responses to the two questions at baseline, 6-month, and 24-month follow-ups and clinically indicated adjuvant therapies (final sample for hormonal therapy n = 3610; chemotherapy n = 2450; radiation therapy = 3258)………………………………………………………………….28

| Supplemental Table 1. Clinical indications of chemotherapy based on National Comprehensive Cancer Network guidelines among breast cancer patients that responded to discrimination due to race or ethnicity domain questions in the Pathways Study (n = 4335) | | | | | | | | | |
| --- | --- | --- | --- | --- | --- | --- | --- | --- | --- |
|  | |  | **HER2** | | | |  | Indication Status | **Total** |
| **Tumor size** | **Nodal status** | **ER/PR** | **0 (Not done)** | **1 (+)** | **2 (-)** | **8 (ordered)** | **Total** | Indicated | 2456 |
| **0 (None)** | **1 (+)** | **0 (Unk)** | 0 | 0 | 0 | 0 | 0 |  |  |
|  |  | **1 (+)** | 0 | 1 | 5 | 0 | 6 |  |  |
|  |  | **2 (-)** | 0 | 1 | 0 | 0 | 1 |  |  |
|  | **2 (-)** | **0 (Unk)** | 0 | 0 | 0 | 0 | 0 |  |  |
|  |  | **1 (+)** | 0 | 0 | 0 | 0 | 0 |  |  |
|  |  | **2 (-)** | 0 | 0 | 0 | 0 | 0 |  |  |
|  | **NA** | **0 (Unk)** | 0 | 0 | 0 | 0 | 0 |  |  |
|  |  | **1 (+)** | 0 | 0 | 0 | 0 | 0 |  |  |
|  |  | **2 (-)** | 0 | 0 | 0 | 0 | 0 |  |  |
| **1 (<= 0.5 cm)** | **1 (+)** | **0 (Unk)** | 0 | 0 | 0 | 0 | 0 |  |  |
|  |  | **1 (+)** | 1 | 1 | 9 | 0 | 11 |  |  |
|  |  | **2 (-)** | 0 | 5 | 0 | 0 | 5 |  |  |
|  | **2 (-)** | **0 (Unk)** | 1 | 0 | 0 | 0 | 1 |  |  |
|  |  | **1 (+)** | 18 | 22 | 168 | 0 | 208 |  |  |
|  |  | **2 (-)** | 2 | 16 | 13 | 0 | 31 |  |  |
|  | **NA** | **0 (Unk)** | 0 | 0 | 0 | 0 | 0 |  |  |
|  |  | **1 (+)** | 0 | 0 | 0 | 0 | 0 |  |  |
|  |  | **2 (-)** | 0 | 0 | 0 | 0 | 0 |  |  |
| **2 (0.6 - 1 cm)** | **1 (+)** | **0 (Unk)** | 0 | 0 | 0 | 0 | 0 |  |  |
|  |  | **1 (+)** | 1 | 6 | 67 | 0 | 74 |  |  |
|  |  | **2 (-)** | 0 | 3 | 3 | 0 | 6 |  |  |
|  | **2 (-)** | **0 (Unk)** | 0 | 0 | 0 | 0 | 0 |  |  |
|  |  | **1 (+)** | 20 | 28 | 407 | 0 | 455 |  |  |
|  |  | **2 (-)** | 2 | 12 | 46 | 0 | 60 |  |  |
|  | **NA** | **0 (Unk)** | 0 | 0 | 0 | 0 | 0 |  |  |
|  |  | **1 (+)** | 0 | 0 | 1 | 0 | 1 |  |  |
|  |  | **2 (-)** | 0 | 0 | 0 | 0 | 0 |  |  |
| **3 (> 1 cm)** | **1 (+)** | **0 (Unk)** | 0 | 0 | 0 | 0 | 0 |  |  |
|  |  | **1 (+)** | 26 | 113 | 651 | 0 | 790 |  |  |
|  |  | **2 (-)** | 3 | 55 | 118 | 0 | 176 |  |  |
|  | **2 (-)** | **0 (Unk)** | 0 | 0 | 0 | 0 | 0 |  |  |
|  |  | **1 (+)** | 40 | 133 | 1032 | 0 | 1205 |  |  |
|  |  | **2 (-)** | 12 | 58 | 223 | 0 | 293 |  |  |
|  | **NA** | **0 (Unk)** | 0 | 0 | 0 | 0 | 0 |  |  |
|  |  | **1 (+)** | 0 | 1 | 6 | 0 | 7 |  |  |
|  |  | **2 (-)** | 0 | 1 | 1 | 0 | 2 |  |  |
| **4 (Microinvasive)** | **1 (+)** | **0 (Unk)** | 0 | 0 | 0 | 0 | 0 |  |  |
|  |  | **1 (+)** | 0 | 0 | 1 | 0 | 1 |  |  |
|  |  | **2 (-)** | 0 | 0 | 0 | 0 | 0 |  |  |
|  | **2 (-)** | **0 (Unk)** | 0 | 0 | 0 | 0 | 0 |  |  |
|  |  | **1 (+)** | 4 | 1 | 12 | 1 | 18 |  |  |
|  |  | **2 (-)** | 2 | 5 | 2 | 0 | 9 |  |  |
|  | **NA** | **0 (Unk)** | 0 | 0 | 0 | 0 | 0 |  |  |
|  |  | **1 (+)** | 0 | 0 | 0 | 0 | 0 |  |  |
|  |  | **2 (-)** | 0 | 0 | 0 | 0 | 0 |  |  |
| **5 (Unknown)** | **1 (+)** | **0 (Unk)** | 0 | 0 | 0 | 0 | 0 |  |  |
|  |  | **1 (+)** | 1 | 4 | 14 | 0 | 19 |  |  |
|  |  | **2 (-)** | 0 | 3 | 3 | 0 | 6 |  |  |
|  | **2 (-)** | **0 (Unk)** | 0 | 0 | 0 | 0 | 0 |  |  |
|  |  | **1 (+)** | 3 | 5 | 36 | 0 | 44 |  |  |
|  |  | **2 (-)** | 0 | 2 | 5 | 0 | 7 |  |  |
|  | **NA** | **0 (Unk)** | 0 | 0 | 0 | 0 | 0 |  |  |
|  |  | **1 (+)** | 0 | 3 | 3 | 0 | 6 |  |  |
|  |  | **2 (-)** | 0 | 2 | 0 | 0 | 2 |  |  |
|  |  |  | 136 | 481 | 2826 | 1 | **3444** |  |  |
| The data shown are for patients under 70 years old. Chemotherapy is discretionary for patients 70 years or older (n = 888). Three participants had an unknown diagnosis age. Of the 2456 participants clinically indicated for receiving chemotherapy, six participants did not have chemotherapy initiation data.  Unk = Unknown | | | | | | | | | |

| Supplemental Table 2. Clinical indications for radiation therapy based on National Comprehensive Cancer Network guidelines among breast cancer patients that responded to discrimination due to race or ethnicity domain questions in the Pathways Study (n = 4335) | | | | | | | | |
| --- | --- | --- | --- | --- | --- | --- | --- | --- |
|  |  |  | **Initiated** | |  |  |  |  |
| **Type of Surgery** | **AJCC Stage** | **Nodal status** | **0/No** | **1/Yes** | **Total** | **Indication Status** |  |  |
| **0/No Surgery** | **1** | **1 (+)** | 1 | 0 | 1 | RT Indicated | 3258 |  |
|  |  | **2 (-)** | 14 | 4 | 18 | RT initiation by indication | 1854 |  |
|  |  | **NA** | 3 | 0 | 3 | RT non-initiation by indication | 1404 |  |
|  | **2** | **1 (+)** | 20 | 1 | 21 |  |  |  |
|  |  | **2 (-)** | 17 | 4 | 21 | RT not indicated | 1077 |  |
|  |  | **NA** | 4 | 0 | 4 |  |  |  |
|  | **3** | **1 (+)** | 26 | 3 | 29 | **Total** | **4335** |  |
|  |  | **2 (-)** | 4 | 0 | 4 |  |  |  |
|  |  | **NA** | 3 | 0 | 3 |  |  |  |
|  | **4** | **1 (+)** | 18 | 2 | 20 |  |  |  |
|  |  | **2 (-)** | 7 | 0 | 7 |  |  |  |
|  |  | **NA** | 7 | 0 | 7 |  |  |  |
| **1/Lumpectomy** | **1** | **1 (+)** | 3 | 1 | 4 |  |  |  |
|  |  | **2 (-)** | 364 | 1307 | 1671 |  |  |  |
|  |  | **NA** | 1 | 1 | 2 |  |  |  |
|  | **2** | **1 (+)** | 240 | 198 | 438 |  |  |  |
|  |  | **2 (-)** | 136 | 196 | 332 |  |  |  |
|  |  | **NA** | 3 | 0 | 3 |  |  |  |
|  | **3** | **1 (+)** | 77 | 19 | 96 |  |  |  |
|  |  | **2 (-)** | 2 | 0 | 2 |  |  |  |
|  |  | **NA** | 0 | 0 | 0 |  |  |  |
|  | **4** | **1 (+)** | 6 | 5 | 11 |  |  |  |
|  |  | **2 (-)** | 1 | 1 | 2 |  |  |  |
|  |  | **NA** | 0 | 0 | 0 |  |  |  |
| **2/Mastectomy** | **1** | **1 (+)** | 5 | 0 | 5 |  |  |  |
|  |  | **2 (-)** | 632 | 11 | 643 |  |  |  |
|  |  | **NA** | 0 | 0 | 0 |  |  |  |
|  | **2** | **1 (+)** | 342 | 41 | 383 |  |  |  |
|  |  | **2 (-)** | 277 | 14 | 291 |  |  |  |
|  |  | **NA** | 2 | 1 | 3 |  |  |  |
|  | **3** | **1 (+)** | 207 | 75 | 282 |  |  |  |
|  |  | **2 (-)** | 3 | 3 | 6 |  |  |  |
|  |  | **NA** | 1 | 0 | 1 |  |  |  |
|  | **4** | **1 (+)** | 11 | 5 | 16 |  |  |  |
|  |  | **2 (-)** | 2 | 1 | 3 |  |  |  |
|  |  | **NA** | 0 | 1 | 1 |  |  |  |
| **3/Surgery, Not Otherwise Specified** | **1** | **1 (+)** | 0 | 0 | 0 |  |  |  |
|  |  | **2 (-)** | 1 | 1 | 2 |  |  |  |
|  |  | **NA** | 0 | 0 | 0 |  |  |  |
|  | **2** | **1 (+)** | 0 | 0 | 0 |  |  |  |
|  |  | **2 (-)** | 0 | 0 | 0 |  |  |  |
|  |  | **NA** | 0 | 0 | 0 |  |  |  |
|  | **3** | **1 (+)** | 0 | 0 | 0 |  |  |  |
|  |  | **2 (-)** | 0 | 0 | 0 |  |  |  |
|  |  | **NA** | 0 | 0 | 0 |  |  |  |
|  | **4** | **1 (+)** | 0 | 0 | 0 |  |  |  |
|  |  | **2 (-)** | 0 | 0 | 0 |  |  |  |
| **Total** |  |  | 2440 | 1895 | 4335 |  |  |  |

Supplemental Table 3. Comparing final vs. excluded samples for hormonal therapy (n = 4504)

|  | Final sample for analysis (n = 3610) | Excluded due to missing in two SRREDPPI questions (n = 169) | Excluded due to missing criteria for hormonal therapy initiation (n = 725) |
| --- | --- | --- | --- |
| Variable | n (column %) | n (column %) | n (column %) |
| SRREDPPI |  |  |  |
| Ever | 461 (12.8) | NA | 126 (17.4) |
| Never | 3149 (87.2) | NA | 599 (82.6) |
| NHW vs. Minorities |  |  |  |
| Non-Hispanic White | 2444 (67.7) | 87 (51.5) | 419 (57.8) |
| Race or ethnic minorities | 1166 (32.3) | 82 (48.5) | 306 (42.2) |
| Race/ethnicity |  |  |  |
| Non-Hispanic White | 2444 (67.7) | 87 (51.5) | 419 (57.8) |
| Black | 215 (6.0) | 24 (14.2) | 112 (15.4) |
| Asian | 475 (13.2) | 22 (13.0) | 89 (12.3) |
| Hispanic | 402 (11.1) | 28 (16.6) | 82 (11.3) |
| American Indian, Alaska Native, and Pacific Islander | 74 (2.0) | 8 (4.7) | 23 (3.2) |
| Country of birth^a^ |  |  |  |
| US-Born | 2861 (79.3) | 126 (76.8) | 572 (78.9) |
| Non-US Born | 745 (20.7) | 38 (23.2) | 153 (21.1) |
| Age at diagnosis^a^ |  |  |  |
| Years, (mean ± SD) | 59.8 ± 12.0 | 57.7 ± 12.5 | 57.2 ± 12.2 |
| Education level |  |  |  |
| High school or less | 557 (15.4) | 31 (18.3) | 119 (16.4) |
| Some college | 1211 (33.5) | 74 (43.8) | 282 (38.9) |
| College graduate | 1010 (28.0) | 36 (21.3) | 195 (26.9) |
| Post graduate | 829 (23.0) | 23 (13.6) | 129 (17.8) |
| Unknown | 3 (0.1) | 5 (3.0) | 0 (0) |
| Income level |  |  |  |
| < $25K | 337 (9.3) | 12 (7.1) | 84 (11.6) |
| $25K – $49K | 685 (19.0) | 25 (14.8) | 134 (18.5) |
| $50K - $89K | 1045 (28.9) | 30 (17.8) | 206 (28.4) |
| ≥ $90K | 1158 (32.1) | 26 (15.4) | 226 (31.2) |
| Unknown | 385 (10.7) | 76 (45.0) | 75 (10.3) |
| Marital status |  |  |  |
| Married/lived as married | 2228 (61.7) | 74 (43.8) | 435 (60.0) |
| Single/separated/widowed | 1371 (38.0) | 90 (53.3) | 290 (40.0) |
| Unknown | 11 (0.3) | 5 (3.0) | 0 (0) |
| AJCC staging |  |  |  |
| I | 2028 (56.2) | 84 (49.7) | 321 (44.3) |
| II | 1199 (33.2) | 61 (36.1) | 297 (41.0) |
| III | 330 (9.1) | 18 (10.7) | 93 (12.8) |
| IV | 53 (1.5) | 6 (3.6) | 14 (1.9) |
| ^a^n = 9 women with missing country of birth  ^b^n = 8 women with missing age of diagnosis | | | |

Supplemental Table 4. Comparing final vs. excluded samples for radiation therapy (n = 4504)

|  | Final sample for analysis (n = 3258) | Excluded due to missing in two SRREDPPI questions (n = 169) | Excluded due to missing criteria for radiation therapy initiation (n = 1077) |
| --- | --- | --- | --- |
| Variable | n (column %) | n (column %) | n (column %) |
| SRREDPPI |  |  |  |
| Ever | 429 (13.2) | NA | 158 (14.7) |
| Never | 2829 (86.8) | NA | 919 (85.3) |
| NHW vs. Minorities |  |  |  |
| Non-Hispanic White | 2197 (67.4) | 87 (51.5) | 666 (61.8) |
| Race or ethnic minorities | 1061 (32.6) | 82 (48.5) | 411 (38.2) |
| Race/ethnicity |  |  |  |
| Non-Hispanic White | 2197 (67.4) | 87 (51.5) | 666 (61.8) |
| Black | 253 (7.8) | 24 (14.2) | 74 (6.9) |
| Asian | 375 (11.5) | 22 (13.0) | 189 (17.5) |
| Hispanic | 355 (10.9) | 28 (16.6) | 129 (12.0) |
| American Indian, Alaska Native, and Pacific Islander | 78 (2.4) | 8 (4.7) | 19 (1.8) |
| Country of birth^a^ |  |  |  |
| US-Born | 2622 (80.6) | 126 (76.8) | 811 (75.4) |
| Non-US Born | 633 (19.4) | 38 (23.2) | 265 (24.6) |
| Age at diagnosis^b^ |  |  |  |
| Years, (mean ± SD) | 59.7 ± 11.8 | 57.7 ± 12.5 | 58.3 ± 12.8 |
| Education level |  |  |  |
| High school or less | 506 (15.5) | 31 (18.3) | 170 (15.8) |
| Some college | 1120 (34.4) | 74 (43.8) | 373 (34.6) |
| College graduate | 882 (27.1) | 36 (21.3) | 323 (30.0) |
| Post graduate | 748 (23.0) | 23 (13.6) | 210 (19.5) |
| Unknown | 2 (0.1) | 5 (3.0) | 1 (0.1) |
| Income level |  |  |  |
| < $25K | 309 (9.5) | 12 (7.1) | 112 (10.4) |
| $25K – $49K | 622 (19.1) | 25 (14.8) | 197 (18.3) |
| $50K - $89K | 958 (29.4) | 30 (17.8) | 293 (27.2) |
| ≥ $90K | 1024 (31.4) | 26 (15.4) | 360 (33.4) |
| Unknown | 345 (10.6) | 76 (45.0) | 115 (10.7) |
| Marital status |  |  |  |
| Married/lived as married | 2013 (61.8) | 74 (43.8) | 650 (60.4) |
| Single/separated/widowed | 1242 (38.1) | 90 (53.3) | 419 (38.9) |
| Unknown | 3 (0.01) | 5 (3.0) | 8 (0.7) |
| AJCC staging |  |  |  |
| I | 1682 (51.6) | 84 (49.7) | 667 (61.9) |
| II | 1156 (35.5) | 61 (36.1) | 340 (31.6) |
| III | 387 (11.9) | 18 (10.7) | 36 (3.3) |
| IV | 33 (1.0) | 6 (3.6) | 34 (3.2) |
| Type of Surgery |  |  |  |
| No Surgery | 0 (0) | 9 (5.3) | 138 (12.8) |
| Lumpectomy | 2561 (78.6) | 94 (55.6) | 0 (0) |
| Mastectomy | 697 (21.4) | 66 (39.1) | 937 (87.0) |
| Surgery, NOS | 0 (0) | 0 (0) | 2 (0.2) |
| ^a^n = 9 women with missing country of birth  ^b^n = 8 women with missing age of diagnosis | | | |

Supplemental Table 5. Comparing final vs. excluded samples for chemotherapy (n = 4504)

|  | Final sample for analysis (n = 2450) | Excluded due to missing in two SRREDPPI questions (n = 169) | Excluded due to missing criteria for chemotherapy initiation  (n = 1885) |
| --- | --- | --- | --- |
| Variable | n (column %) | n (column %) | n (column %) |
| SRREDPPI |  |  |  |
| Ever | 346 (14.1) | NA | 241 (12.8) |
| Never | 2104 (85.9) | NA | 1644 (87.2) |
| NHW vs. Minorities |  |  |  |
| Non-Hispanic White | 1498 (61.1) | 87 (51.5) | 1365 (72.4) |
| Race or ethnic minorities | 952 (38.9) | 82 (48.5) | 520 (27.6) |
| Race/ethnicity |  |  |  |
| Non-Hispanic White | 1498 (61.1) | 87 (51.5) | 1365 (72.4) |
| Black | 212 (8.7) | 24 (14.2) | 115 (6.1) |
| Asian | 381 (15.6) | 22 (13.0) | 183 (9.7) |
| Hispanic | 298 (12.2) | 28 (16.6) | 186 (9.9) |
| American Indian, Alaska Native, and Pacific Islander | 61 (2.5) | 8 (4.7) | 36 (1.9) |
| Country of birth^a^ |  |  |  |
| US-Born | 1879 (76.7) | 126 (76.8) | 1554 (82.6) |
| Non-US Born | 571 (23.3) | 38 (23.2) | 327 (17.4) |
| Age at diagnosis^b^ |  |  |  |
| Years, (mean ± SD) | 54.4 ± 9.4 | 57.7 ± 12.5 |  |
| Education level |  |  |  |
| High school or less | 350 (14.3) | 31 (18.3) | 326 (17.3) |
| Some college | 836 (34.1) | 74 (43.8) | 657 (34.9) |
| College graduate | 710 (29.0) | 36 (21.3) | 495 (26.3) |
| Post graduate | 554 (22.6) | 23 (13.6) | 404 (21.4) |
| Unknown | 0 (0) | 5 (3.0) | 3 (0.2) |
| Income level |  |  |  |
| < $25K | 160 (6.5) | 12 (7.1) | 261 (13.8) |
| $25K – $49K | 415 (16.9) | 25 (14.8) | 404 (21.4) |
| $50K - $89K | 750 (30.6) | 30 (17.8) | 501 (26.6) |
| ≥ $90K | 895 (36.5) | 26 (15.4) | 489 (25.9) |
| Unknown | 230 (9.4) | 76 (45.0) | 230 (12.2) |
| Marital status |  |  |  |
| Married/lived as married | 1604 (65.5) | 74 (43.8) | 1059 (56.2) |
| Single/separated/widowed | 843 (34.4) | 90 (53.3) | 818 (43.4) |
| Unknown | 3 (0.1) | 5 (3.0) | 8 (0.4) |
| AJCC staging |  |  |  |
| I | 947 (38.7) | 84 (49.7) | 1402 (74.4) |
| II | 1139 (46.5) | 61 (36.1) | 357 (18.9) |
| III | 327 (13.3) | 18 (10.7) | 96 (5.1) |
| IV | 37 (1.5) | 6 (3.6) | 30 (1.6) |
| HR Status |  |  |  |
| Positive (3759) | 1997 (81.5) | 125 (74.0) | 1637 (86.8) |
| Negative (745) | 453 (18.5) | 44 (26.0) | 248 (13.2) |
| HER2 Status |  |  |  |
| Not Done | 0 (0) | 3 (1.8) | 182 (9.7) |
| Positive | 365 (14.9) | 28 (16.6) | 195 (10.3) |
| Negative | 2085 (85.1) | 138 (81.7) | 1507 (79.9) |
| Order/Not Applicable | 0 (0) | 0 (0) | 1 (0.1) |
| ^a^n = 9 women with missing country of birth  ^b^n = 8 women with missing age of diagnosis | | | |

| Supplemental Table 6. Distribution of demographic variables stratified by ever vs. never self-reported racial or ethnic discrimination in patient-provider interactions among chemotherapy indicated breast cancer patients (n = 2450) | | | | |
| --- | --- | --- | --- | --- |
|  | Total  (n = 2450) | Ever SRREDPPI  (n = 346) | Never SRREDPPI  (n = 2104) |  |
| Variable | n (column %) | n (column %) | n (column %) | P for difference |
| NHW vs. Minority |  |  |  | <0.0001 |
| Non-Hispanic White | 1498 (61.1) | 91 (26.3) | 1407 (66.9) |  |
| Racial or ethnic minority | 952 (38.9) | 255 (73.7) | 697 (33.1) |  |
| Race and ethnicity strata |  |  |  | <0.0001 |
| Non-Hispanic White | 1498(61.1) | 91 (26.3) | 1407 (66.9) |  |
| Black | 212 (8.7) | 71 (20.5) | 141 (6.7) |  |
| Asian | 381 (15.6) | 120 (34.7) | 261 (12.4) |  |
| Hispanic | 298 (12.2) | 56 (16.2) | 242 (11.5) |  |
| American Indian, Alaska Native, or Pacific Islander | 61 (2.5) | 8 (2.3) | 53 (2.5) |  |
| Country of birth |  |  |  | <0.0001 |
| US-Born | 1879 (76.7) | 198 (57.2) | 1681 (79.9) |  |
| Non-US Born | 571 (23.3) | 148 (42.8) | 423 (20.1) |  |
| Age at diagnosis |  |  |  | 0.040 |
| Years, (mean ± SD) | 54.4 ± 9.4 | 53.5 ± 9.3 | 54.9 ± 9.4 |  |
| Education level |  |  |  | 0.23 |
| High school or less | 350 (14.3) | 50 (14.5) | 300 (14.3) |  |
| Some college | 836 (34.1) | 121 (35.0) | 715 (34.0) |  |
| College graduate | 710 (29.0) | 113 (32.7) | 597 (28.4) |  |
| Post graduate | 554 (22.6) | 62 (17.9) | 492 (23.4) |  |
| Income level |  |  |  | 0.0003 |
| < $25K | 160 (6.5) | 29 (8.4) | 131 (6.2) |  |
| $25K – $49K | 415 (16.9) | 80 (23.1) | 335 (15.9) |  |
| $50K - $89K | 750 (30.6) | 107 (30.9) | 643 (30.6) |  |
| ≥ $90K | 895 (36.5) | 101 (29.2) | 794 (37.7) |  |
| Unknown | 230 (9.4) | 29 (8.4) | 201 (9.6) |  |
| Marital status |  |  |  | 0.005 |
| Married/lived as married | 1604 (65.5) | 204 (59.0) | 1400 (66.5) |  |
| Single/separated/widowed | 843 (34.4) | 141 (40.8) | 702 (33.4) |  |
| Unknown | 3 (0.1) | 1 (0.2) | 2 (0.1) |  |
| AJCC staging |  |  |  | 0.80 |
| I | 947 (38.7) | 128 (37.0) | 819 (38.9) |  |
| II | 1139 (46.5) | 166 (48.0) | 973 (46.2) |  |
| III | 327 (13.3) | 50 (14.5) | 277 (13.2) |  |
| IV | 37 (1.5) | 2 (0.6) | 35 (1.7) |  |
| HR Status |  |  |  | 0.003 |
| Positive | 1997 (81.5) | 262 (75.7) | 1735 (82.5) |  |
| Negative | 453 (18.5) | 84 (24.3) | 369 (17.5) |  |
| HER2 |  |  |  | 0.72 |
| Positive | 365 (14.9) | 50 (14.5) | 315 (15.0) |  |
| Negative | 2085 (85.1) | 296 (85.5) | 1789 (85.0) |  |
| SRREDPPI = self-reported racial/ethnic discrimination in patient-provider interactions  NHW = Non-Hispanic White  SD = standard deviation  AJCC = American Joint Committee on Cancer  P for difference indicates descriptive univariate analyses comparing the non-initiation and initiation of chemotherapy examining demographics and clinical categorical variables | | | | |

| Supplemental Table 7. Distribution of demographic variables stratified by ever vs. never self-reported racial or ethnic discrimination in patient-provider interactions among radiation therapy indicated breast cancer patients (n = 3258) | | | | |
| --- | --- | --- | --- | --- |
|  | Total  (n = 3258) | Ever SRREDPPI  (n = 429) | Never SRREDPPI  (n = 2829) |  |
| Variable | n (column %) | n (column %) | n (column %) | P for difference |
| NHW vs. Minorities |  |  |  | <0.0001 |
| Non-Hispanic White | 2197 (67.4) | 145 (33.8) | 2052 (72.5) |  |
| Race or ethnic minorities | 1061 (32.6) | 284 (66.2) | 777 (27.5) |  |
| Race and ethnicity |  |  |  | <0.0001 |
| Non-Hispanic White | 2197 (67.4) | 145 (33.8) | 2052 (72.5) |  |
| Black | 253 (7.8) | 91 (21.2) | 162 (5.7) |  |
| Asian | 375 (11.5) | 118 (27.5) | 257 (9.1) |  |
| Hispanic | 355 (10.9) | 65 (15.2) | 290 (10.3) |  |
| American Indian, Alaska Native, and Pacific Islander | 78 (2.4) | 10 (2.3) | 68 (2.4) |  |
| Country of birth^a^ |  |  |  | <0.0001 |
| US-Born | 2622 (80.6) | 282 (65.7) | 2340 (82.8) |  |
| Non-US Born | 633 (19.4) | 147 (34.3) | 486 (17.2) |  |
| Age at diagnosis^b^ |  |  |  | 0.001 |
| Years, (mean ± SD) | 59.7 ± 11.8 | 58.0 ± 12.0 | 60.0 ± 11.8 |  |
| Education level |  |  |  | 0.83 |
| High school or less | 506 (15.5) | 63 (14.7) | 443 (15.7) |  |
| Some college | 1120 (34.4) | 150 (35.0) | 970 (34.3) |  |
| College graduate | 882 (27.1) | 126 (29.4) | 756 (26.7) |  |
| Post graduate | 748 (23.0) | 90 (21.0) | 658 (23.3) |  |
| Unknown | 2 (0.1) | 0 (0) | 2 (0.1) |  |
| Income level |  |  |  | <0.001 |
| < $25K | 309 (9.5) | 46 (10.7) | 263 (9.3) |  |
| $25K – $49K | 622 (19.1) | 108 (25.2) | 514 (18.2) |  |
| $50K - $89K | 958 (29.4) | 125 (29.1) | 833 (29.4) |  |
| ≥ $90K | 1024 (31.4) | 108 (25.2) | 916 (32.4) |  |
| Unknown | 345 (10.6) | 42 (9.8) | 303 (10.7) |  |
| Marital status |  |  |  | 0.002 |
| Married/lived as married | 2013 (61.8) | 235 (54.8) | 1778 (62.8) |  |
| Single/separated/widowed | 1242 (38.1) | 194 (45.2) | 1048 (37.0) |  |
| Unknown | 3 (0.01) | 0 (0) | 3 (0.1) |  |
| AJCC staging |  |  |  | 0.84 |
| I | 1682 (51.6) | 215 (50.1) | 1467 (51.9) |  |
| II | 1156 (35.5) | 159 (37.1) | 997 (35.2) |  |
| III | 387 (11.9) | 54 (12.6) | 333 (11.8) |  |
| IV | 33 (1.0) | 1 (0.2) | 32 (1.1) |  |
| Surgery |  |  |  | 0.51 |
| Lumpectomy | 2561 (78.6) | 332 (77.4) | 2229 (78.8) |  |
| Mastectomy | 697 (21.4) | 97 (22.6) | 600 (21.2) |  |
| ^a^n = 3 women with missing country of birth.  ^b^n = 2 women with missing age at diagnosis.  SRREDPPI = self-reported racial/ethnic discrimination in patient-provider interactions  NHW = Non-Hispanic White  SD = standard deviation  AJCC = American Joint Committee on Cancer  P for difference indicates descriptive univariate analyses comparing the non-initiation and initiation of radiation therapy examining demographics and clinical categorical variables | | | | |

| Supplemental Table 8. Distribution of demographic variables stratified by ever vs. never self-reported racial or ethnic discrimination in patient-provider interactions among HR-positive breast cancer patients (n = 3,610) | | | | |
| --- | --- | --- | --- | --- |
|  | Total  (n = 3610) | Ever SRREDPPI  (n = 461) | Never SRREDPPI  (n = 3149) |  |
| Variable | n (column %) | n (column %) | n (column %) | P for difference |
| NHW vs. Minority |  |  |  | <0.0001 |
| Non-Hispanic White | 2444 (67.7) | 173 (37.5) | 2271 (72.1) |  |
| Racial or ethnic minority | 1166 (32.3) | 288 (62.5) | 878 (27.9) |  |
| Race and ethnicity strata |  |  |  | <0.0001 |
| Non-Hispanic White | 2444 (67.7) | 173 (37.5) | 2271 (72.1) |  |
| Black | 215 (6.0) | 74 (16.1) | 141 (4.5) |  |
| Asian | 475 (13.2) | 141 (30.6) | 334 (10.6) |  |
| Hispanic | 402 (11.1) | 65 (14.1) | 337 (10.7) |  |
| American Indian, Alaska Native, and Pacific Islander | 74 (2.0) | 8 (1.7) | 66 (2.1) |  |
| Country of birth^a^ |  |  |  | <0.0001 |
| US-Born | 2861 (79.3) | 284 (61.7) | 2577 (81.9) |  |
| Non-US Born | 745 (20.7) | 176 (38.3) | 569 (18.1) |  |
| Age at diagnosis^b^ |  |  |  | 0.003 |
| Years, (mean ± SD) | 59.8 ± 12.0 | 58.3 ± 12.1 | 60.0 ± 12.0 |  |
| Education level |  |  |  | 0.82 |
| High school or less | 557 (15.4) | 71 (15.4) | 486 (15.4) |  |
| Some college | 1211 (33.5) | 152 (33.0) | 1059 (33.6) |  |
| College graduate | 1010 (28.0) | 140 (30.4) | 870 (27.6) |  |
| Post graduate | 829 (23.0) | 97 (21.0) | 732 (23.2) |  |
| Unknown | 3 (0.1) | 1 (0.2) | 2 (0.1) |  |
| Income level |  |  |  | 0.021 |
| < $25K | 337 (9.3) | 48 (10.4) | 289 (9.2) |  |
| $25K – $49K | 685 (19.0) | 106 (23.0) | 579 (18.4) |  |
| $50K - $89K | 1045 (28.9) | 132 (28.6) | 913 (29.0) |  |
| ≥ $90K | 1158 (32.1) | 128 (27.8) | 1030 (32.7) |  |
| Unknown | 385 (10.7) | 47 (10.2) | 338 (10.7) |  |
| Marital status |  |  |  | 0.001 |
| Married/lived as married | 2228 (61.7) | 253 (54.9) | 1975 (62.7) |  |
| Single/separated/widowed | 1371 (38.0) | 206 (44.7) | 1165 (37.0) |  |
| Unknown | 11 (0.3) | 2 (0.4) | 9 (0.3) |  |
| AJCC staging |  |  |  | 0.85 |
| I | 2028 (56.2) | 259 (56.2) | 1769 (56.2) |  |
| II | 1199 (33.2) | 153 (33.2) | 1046 (33.2) |  |
| III | 330 (9.1) | 45 (9.8) | 285 (9.1) |  |
| IV | 53 (1.5) | 4 (0.9) | 49 (1.6) |  |
| ^a^ n = 4 women with missing country of origin.  ^b^ n = 3 women with missing age at diagnosis.  SRREDPPI = Self-reported racial/ethnic discrimination in patient-provider interactions  NHW = Non-Hispanic White  SD = standard deviation  AJCC = American Joint Committee on Cancer  P for difference indicates descriptive univariate analyses comparing the non-initiation and initiation of hormonal therapy examining demographics and clinical categorical variables | | | | |

| Supplemental Table 9. Results of univariable and multivariable logistic regression analyses for ever/never self-reports of racial or ethnic discrimination in patient-provider interactions (SRREDPPI) and hormonal therapy initiation by type of analysis and race and ethnicity-stratified subpopulations among HR-positive breast cancer patients in the Pathways Study (n = 3,554) **(Baseline only)** | | | | | |
| --- | --- | --- | --- | --- | --- |
| Type of Analysis | SRREDPPI | Hormonal Therapy Initiation | | Univariable Models | Multivariable Models^a^ |
|  |  | No | Yes | OR (95% CI) | aOR (95% CI) |
|  |  | n (col %) | n (col %) |  |  |
| Two-category race and ethnicity variable | Ever | 65 (16.5) | 390 (12.3) | 1.41 (1.05 – 1.86) | 1.44 (1.06 – 1.94) |
|  | Never | 328 (83.5) | 2771 (87.7) |  |  |
| Five-category race and ethnicity variable | Ever | 65 (16.5) | 390 (12.3) | 1.41 (1.05 – 1.86) | 1.51 (1.10 – 2.05) |
|  | Never | 328 (83.5) | 2771 (87.7) |  |  |
| Non-Hispanic White | Ever | 27 (9.9) | 143 (6.7) | 1.54 (0.98 – 2.33) | 1.41 (0.89 – 2.17) |
|  | Never | 245 (90.1) | 1993 (93.3) |  |  |
| Racial or ethnic minority combined | Ever | 38 (31.4) | 247 (24.1) | 1.44 (0.95 – 2.16) | 1.41 (0.91 – 2.14) |
|  | Never | 83 (68.6) | 778 (75.9) |  |  |
| Black | Ever | 10 (50.0) | 64 (33.9) | 1.95 (0.76 – 5.00) | 1.62 (0.60 – 4.39) |
|  | Never | 10 (50.0) | 125 (66.1) |  |  |
| Asian | Ever | 15 (35.7) | 124 (29.2) | 1.35 (0.68 – 2.59) | 1.32 (0.64 – 2.65) |
|  | Never | 27 (64.3) | 301 (70.8) |  |  |
| Hispanic | Ever | 10 (22.2) | 54 (15.4) | 1.57 (0.70 – 3.26) | 1.65 (0.71 – 3.57) |
|  | Never | 35 (77.8) | 297 (84.6) |  |  |
| American Indian, Alaska Native, Pacific Islander | Ever | — | — | 3.00 (0.55 – 14.19) | 20.87 (1.66 – 414.48) |
|  | Never | 11 (78.6) | 55 (91.7) |  |  |
| ^a^Adjusted for covariates including race or ethnicity (primary: NHW vs. WOC; sub-analysis: race or ethnicity strata), country of birth, age, education level, income level, marital status, and AJCC staging.  SRREDPPI = Self-reported racial/ethnic discrimination in patient-provider interactions  OR = odds ratio  aOR = adjusted odds ratio  Two-category race and ethnicity variable = examined the association of non-initiation of hormonal therapy by comparing ever versus never reported discrimination, adjusting for covariates including combined racial or ethnic minority women versus Non-Hispanic White women  Five-category race and ethnicity variable = examined the association of non-initiation of hormonal therapy by comparing ever versus never reported discrimination, adjusted the stratification by each racial/ethnic group of women with Non-Hispanic White women as the reference  To protect patient confidentiality and comply with institutional privacy policies, cell counts ≤5 are not reported and are indicated by '—' in tables. | | | | | |

| Supplemental Table 10. Results of univariable and multivariable logistic regression analyses for ever/never self-reports of racial or ethnic discrimination in patient-provider interactions (SRREDPPI) and hormonal therapy initiation by type of analysis and race and ethnicity-stratified subpopulations among HR-positive breast cancer patients in the Pathways Study (n = 3,602) **(Baseline and 6-month follow-up)** | | | | | |
| --- | --- | --- | --- | --- | --- |
| Type of Analysis | SRREDPPI | Hormonal Therapy Initiation | | Univariable Models | Multivariable Models^a^ |
|  |  | No | Yes | OR (95% CI) | aOR (95% CI) |
|  |  | n (col %) | n (col %) |  |  |
| Two-category race and ethnicity variable | Ever | 65 (16.2) | 393 (12.3) | 1.38 (1.03 – 1.82) | 1.42 (1.04 – 1.91) |
|  | Never | 337 (83.8) | 2807 (87.7) |  |  |
| Five-category race and ethnicity variable | Ever | 65 (16.2) | 393 (12.3) | 1.38 (1.03 – 1.82) | 1.49 (1.09 – 2.02) |
|  | Never | 337 (83.8) | 2807 (87.7) |  |  |
| Non-Hispanic White | Ever | 27 (9.7) | 144 (6.7) | 1.50 (0.96 – 2.27) | 1.39 (0.87 – 2.13) |
|  | Never | 252 (90.3) | 2016 (93.3) |  |  |
| Racial or ethnic minority combined | Ever | 38 (30.9) | 249 (23.9) | 1.42 (0.94 – 2.12) | 1.38 (0.90 – 2.10) |
|  | Never | 85 (69.1) | 791 (76.1) |  |  |
| Black | Ever | 10 (50.0) | 64 (33.0) | 2.03 (0.80 – 5.19) | 1.64 (0.61 – 4.44) |
|  | Never | 10 (50.0) | 130 (67.0) |  |  |
| Asian | Ever | 15 (34.9) | 126 (29.2) | 1.30 (0.65 – 2.48) | 1.26 (0.61 – 2.52) |
|  | Never | 28 (65.1) | 305 (70.8) |  |  |
| Hispanic | Ever | 10 (21.7) | 54 (15.2) | 1.55 (0.69 – 3.20) | 1.66 (0.72 – 3.58) |
|  | Never | 36 (78.3) | 301 (84.8) |  |  |
| American Indian, Alaska Native, Pacific Islander | Ever | — | — | 3.00 (0.55 – 14.19) | 20.87 (1.66 – 414.48) |
|  | Never | 11 (78.6) | 55 (91.7) |  |  |
| ^a^Adjusted for covariates including race or ethnicity (primary: NHW vs. WOC; sub-analysis: race or ethnicity strata), country of birth, age, education level, income level, marital status, and AJCC staging.  SRREDPPI = Self-reported racial/ethnic discrimination in patient-provider interactions  OR = odds ratio  aOR = adjusted odds ratio  Two-category race and ethnicity variable = examined the association of non-initiation of hormonal therapy by comparing ever versus never reported discrimination, adjusting for covariates including combined racial or ethnic minority women versus Non-Hispanic White women  Five-category race and ethnicity variable = examined the association of non-initiation of hormonal therapy by comparing ever versus never reported discrimination, adjusted the stratification by each racial/ethnic group of women with Non-Hispanic White women as the reference  To protect patient confidentiality and comply with institutional privacy policies, cell counts ≤5 are not reported and are indicated by '—' in tables. | | | | | |

| Supplemental Table 11. Results of univariable and multivariable logistic regression analyses for ever/never self-reported racial or ethnic discrimination in patient-provider interactions (SRREDPPI) and radiation therapy initiation by type of analysis and race and ethnicity-stratified subpopulations among radiation therapy indicated breast cancer patients in the Pathways Study (n = 3,202) **(Baseline only)** | | | | | |
| --- | --- | --- | --- | --- | --- |
| Type of Analysis | SRREDPPI | Radiation Therapy Initiation | | Univariable Models | Multivariable Models^a^ |
|  |  | No | Yes | OR (95% CI) | aOR (95% CI) |
|  |  | n (col %) | n (col %) |  |  |
| Two-category race and ethnicity variable | Ever | 209 (15.2) | 213 (11.6) | 1.36 (1.11 – 1.68) | 1.33 (1.04 – 1.70) |
|  | Never | 1163 (84.8) | 1617 (88.4) |  |  |
| Five-category race and ethnicity variable | Ever | 209 (15.2) | 213 (11.6) | 1.36 (1.11 – 1.68) | 1.28 (1.00 – 1.64) |
|  | Never | 1163 (84.8) | 1617 (88.4) |  |  |
| Non-Hispanic White | Ever | 59 (6.8) | 83 (6.4) | 1.08 (0.76 – 1.52) | 1.24 (0.83 – 1.84) |
|  | Never | 804 (93.2) | 1218 (93.6) |  |  |
| Race or ethnic minority combined | Ever | 150 (29.5) | 130 (24.6) | 1.28 (0.97 – 1.69) | 1.39 (1.01 – 1.91) |
|  | Never | 359 (70.5) | 399 (75.4) |  |  |
| Black | Ever | 45 (36.6) | 46 (37.7) | 0.95 (0.57 – 1.60) | 0.95 (0.52 – 1.74) |
|  | Never | 78 (63.4) | 76 (62.3) |  |  |
| Asian | Ever | 66 (34.4) | 50 (28.4) | 1.32 (0.85 – 2.06) | 1.44 (0.83 – 2.50) |
|  | Never | 126 (65.6) | 126 (71.6) |  |  |
| Hispanic | Ever | 32 (20.5) | 31 (16.2) | 1.33 (0.77 – 2.31) | 1.82 (0.95 – 3.54) |
|  | Never | 124 (79.5) | 160 (83.8) |  |  |
| American Indian, Alaska Native, and Pacific Islander | Ever | 7 (18.4) | — | 2.78 (0.71 – 13.77) | 3.41 (0.41 – 50.94) |
|  | Never | 31 (81.6) | 37 (92.5) |  |  |
| ^a^Adjusted for covariates including race or ethnicity (two-category race and ethnicity variable: Non-Hispanic White vs. racial or ethnic minority women combined; five-category race and ethnicity variable: race or ethnicity strata), country of birth, age at diagnosis, education level, income level, marital status, American Joint Committee on Cancer staging and type of surgery.  SRREDPPI = self-reported racial/ethnic discrimination in patient-provider interactions  OR = odds ratio  aOR = adjusted odds ratio  Two-category race and ethnicity variable = examined the association of non-initiation of adjuvant therapies by comparing ever versus never reported discrimination, adjusting for combined racial or ethnic minority women versus NHW women  Five-category race and ethnicity variable = examined the association of non-initiation of adjuvant therapies by comparing ever versus never reported discrimination, adjusting the stratification by each racial/ethnic group of women with NHW women as the reference  To protect patient confidentiality and comply with institutional privacy policies, cell counts ≤5 are not reported and are indicated by '—' in tables | | | | | |

| Supplemental Table 12. Results of univariable and multivariable logistic regression analyses for ever/never self-reported racial or ethnic discrimination in patient-provider interactions (SRREDPPI) and radiation therapy initiation by type of analysis and race and ethnicity-stratified subpopulations among radiation therapy indicated breast cancer patients in the Pathways Study (n = 3,247) **(Baseline and 6-month)** | | | | | |
| --- | --- | --- | --- | --- | --- |
| Type of Analysis | SRREDPPI | Radiation Therapy Initiation | | Univariable Models | Multivariable Models^a^ |
|  |  | No | Yes | OR (95% CI) | aOR (95% CI) |
|  |  | n (col %) | n (col %) |  |  |
| Two-category race and ethnicity variable | Ever | 211 (15.1) | 215 (11.6) | 1.35 (1.10 – 1.65) | 1.28 (1.00 – 1.64) |
|  | Never | 1188 (84.9) | 1633 (88.4) |  |  |
| Five-category race and ethnicity variable | Ever | 211 (15.1) | 215 (11.6) | 1.35 (1.10 – 1.65) | 1.24 (0.97 – 1.59) |
|  | Never | 1188 (84.9) | 1633 (88.4) |  |  |
| Non-Hispanic White | Ever | 59 (6.7) | 84 (6.4) | 1.05 (0.74 – 1.48) | 1.20 (0.81 – 1.77) |
|  | Never | 819 (93.3) | 1229 (93.6) |  |  |
| Race or ethnic minority combined | Ever | 152 (29.2) | 131 (24.5) | 1.27 (0.97 – 1.67) | 1.35 (0.98 – 1.86) |
|  | Never | 369 (70.8) | 404 (75.5) |  |  |
| Black | Ever | 45 (34.9) | 46 (37.7) | 0.89 (0.53 – 1.48) | 0.91 (0.50 – 1.66) |
|  | Never | 84 (65.1) | 76 (62.3) |  |  |
| Asian | Ever | 67 (34.5) | 51 (28.3) | 1.33 (0.86 – 2.08) | 1.41 (0.82 – 2.44) |
|  | Never | 127 (65.5) | 129 (71.7) |  |  |
| Hispanic | Ever | 33 (20.6) | 31 (16.1) | 1.36 (0.79 – 2.34) | 1.75 (0.92 – 3.37) |
|  | Never | 127 (79.4) | 162 (83.9) |  |  |
| American Indian, Alaska Native, and Pacific Islander | Ever | 7 (18.4) | — | 2.78 (0.71 – 13.77) | 3.41 (0.41 – 50.94) |
|  | Never | 31 (81.6) | 37 (92.5) |  |  |
| ^a^Adjusted for covariates including race or ethnicity (two-category race and ethnicity variable: Non-Hispanic White vs. racial or ethnic minority women combined; five-category race and ethnicity variable: race or ethnicity strata), country of birth, age at diagnosis, education level, income level, marital status, American Joint Committee on Cancer staging, and type of surgery.  SRREDPPI = self-reported racial/ethnic discrimination in patient-provider interactions  OR = odds ratio  aOR = adjusted odds ratio  Two-category race and ethnicity variable = examined the association of non-initiation of adjuvant therapies by comparing ever versus never reported discrimination, adjusting for combined racial or ethnic minority women versus NHW women  Five-category race and ethnicity variable = examined the association of non-initiation of adjuvant therapies by comparing ever versus never reported discrimination, adjusting the stratification by each racial/ethnic group of women with NHW women as the reference  To protect patient confidentiality and comply with institutional privacy policies, cell counts ≤5 are not reported and are indicated by '—' in tables. | | | | | |

| Supplemental Table 13. Results of univariable and multivariable logistic regression analyses for ever/never self-reported racial or ethnic discrimination in patient-provider interactions (SRREDPPI) and chemotherapy initiation by type of analysis and race and ethnicity-stratified subpopulations among chemotherapy indicated breast cancer patients in the Pathways Study (n = 2412) **(Baseline only)** | | | | | |
| --- | --- | --- | --- | --- | --- |
| Type of Analysis | SRREDPPI | Chemotherapy Initiation | | Univariable Models | Multivariable Models^a^ |
|  |  | No | Yes | OR (95% CI) | aOR (95% CI) |
|  |  | n (col %) | n (col %) |  |  |
| Two-category race and ethnicity variable | Ever | 99 (12.7) | 244 (14.9) | 0.83 (0.64 – 1.06) | 0.97 (0.69 – 1.36) |
|  | Never | 679 (87.3) | 1390 (85.1) |  |  |
| Five-category race and ethnicity variable | Ever | 99 (12.7) | 244 (14.9) | 0.83 (0.64 – 1.06) | 0.95 (0.68 – 1.33) |
|  | Never | 679 (87.3) | 1390 (85.1) |  |  |
| Non-Hispanic White | Ever | 35 (6.7) | 54 (5.7) | 1.19 (0.76 – 1.84) | 1.34 (0.76 – 2.35) |
|  | Never | 488 (93.3) | 899 (94.3) |  |  |
| Race or ethnic minority combined | Ever | 64 (25.1) | 190 (27.9) | 0.87 (0.62 – 1.20) | 0.78 (0.50 – 1.18) |
|  | Never | 191 (74.9) | 491 (72.1) |  |  |
| Black | Ever | 21 (35.0) | 50 (34.5) | 1.02 (0.54 – 1.91) | 0.90 (0.40 – 1.99) |
|  | Never | 39 (65.0) | 95 (65.5) |  |  |
| Asian | Ever | 24 (23.5) | 95 (34.7) | 0.58 (0.34 – 0.96) | 0.56 (0.28 – 1.09) |
|  | Never | 78 (76.5) | 179 (65.3) |  |  |
| Hispanic | Ever | 18 (22.5) | 38 (17.8) | 1.34 (0.70 – 2.50) | 1.01 (0.37 – 2.74) |
|  | Never | 62 (77.5) | 176 (82.2) |  |  |
| American Indian, Alaska Native, Pacific Islander | Ever | — | 7 (14.6) | 0.49 (0.02 – 3.15) | 2.95 (0.04 – 143.77) |
|  | Never | 12 (92.3) | 41 (85.4) |  |  |
| ^a^Adjusted for covariates including race or ethnicity (two-category race and ethnicity variable: Non-Hispanic White vs. racial or ethnic minority women combined; five-category race and ethnicity variable: race or ethnicity strata), country of birth, age, education level, income level, marital status, American Joint Committee on Cancer staging, hormone receptor (HR) status, and human epidermal growth factor receptor 2 (HER2) status.  SRREDPPI = Self-reported racial/ethnic discrimination in patient-provider interactions  OR = odds ratio  aOR = adjusted odds ratio  Two-category race and ethnicity variable = examined the association of non-initiation of adjuvant therapies by comparing ever versus never reported discrimination, adjusting for combined racial or ethnic minority women versus Non-Hispanic White women  Five-category race and ethnicity variable = examined the association of non-initiation of adjuvant therapies by comparing ever versus never reported discrimination, adjusting the stratification by each racial/ethnic group of women with Non-Hispanic White women as the reference  To protect patient confidentiality and comply with institutional privacy policies, cell counts ≤5 are not reported and are indicated by '—' in tables. | | | | | |

| Supplemental Table 14. Results of univariable and multivariable logistic regression analyses for ever/never self-reported racial or ethnic discrimination in patient-provider interactions (SRREDPPI) and chemotherapy initiation by type of analysis and race and ethnicity-stratified subpopulations among chemotherapy indicated breast cancer patients in the Pathways Study (n = 2441) **(Baseline and 6-month)** | | | | | |
| --- | --- | --- | --- | --- | --- |
| Type of Analysis | SRREDPPI | Chemotherapy Initiation | | Univariable Models | Multivariable Models^a^ |
|  |  | No | Yes | OR (95% CI) | aOR (95% CI) |
|  |  | n (col %) | n (col %) |  |  |
| Two-category race and ethnicity variable | Ever | 99 (12.6) | 246 (14.9) | 0.83 (0.64 – 1.06) | 0.97 (0.69 – 1.35) |
|  | Never | 687 (87.4) | 1409 (85.1) |  |  |
| Five-category race and ethnicity variable | Ever | 99 (12.6) | 246 (14.9) | 0.83 (0.64 – 1.06) | 0.95 (0.68 – 1.33) |
|  | Never | 687 (87.4) | 1409 (85.1) |  |  |
| Non-Hispanic White | Ever | 35 (6.6) | 55 (5.7) | 1.17 (0.75 – 1.81) | 1.29 (0.73 – 2.25) |
|  | Never | 494 (93.4) | 909 (94.3) |  |  |
| Race or ethnic minority combined | Ever | 64 (24.9) | 191 (27.6) | 0.86 (0.62 – 1.20) | 0.78 (0.51 – 1.19) |
|  | Never | 193 (75.1) | 500 (72.4) |  |  |
| Black | Ever | 21 (35.0) | 50 (33.3) | 1.08 (0.57 – 2.01) | 0.93 (0.42 – 2.07) |
|  | Never | 39 (65.0) | 100 (66.7) |  |  |
| Asian | Ever | 24 (23.3) | 96 (34.7) | 0.57 (0.34 – 0.95) | 0.56 (0.28 – 1.08) |
|  | Never | 79 (76.7) | 181 (65.3) |  |  |
| Hispanic | Ever | 18 (22.2) | 38 (17.6) | 1.34 (0.70 – 2.49) | 1.05 (0.39 – 2.81) |
|  | Never | 63 (77.8) | 178 (82.4) |  |  |
| American Indian, Alaska Native, Pacific Islander | Ever | — | 7 (14.6) | 0.49 (0.02 – 3.15) | 2.95 (0.04 – 143.77) |
|  | Never | 12 (92.3) | 41 (85.4) |  |  |
| ^a^Adjusted for covariates including race or ethnicity (two-category race and ethnicity variable: Non-Hispanic White vs. racial or ethnic minority women combined; five-category race and ethnicity variable: race or ethnicity strata), country of birth, age, education level, income level, marital status, American Joint Committee on Cancer staging, hormone receptor (HR) status, and human epidermal growth factor receptor 2 (HER2) status.  SRREDPPI = Self-reported racial/ethnic discrimination in patient-provider interactions  OR = odds ratio  aOR = adjusted odds ratio  Two-category race and ethnicity variable = examined the association of non-initiation of adjuvant therapies by comparing ever versus never reported discrimination, adjusting for combined racial or ethnic minority women versus Non-Hispanic White women  Five-category race and ethnicity variable = examined the association of non-initiation of adjuvant therapies by comparing ever versus never reported discrimination, adjusting the stratification by each racial/ethnic group of women with Non-Hispanic White women as the reference  To protect patient confidentiality and comply with institutional privacy policies, cell counts ≤5 are not reported and are indicated by '—' in tables. | | | | | |

| Supplemental Table 15. Results of univariable and multivariable logistic regression analyses for ever/never self-reports of racial or ethnic discrimination in patient-provider interactions (SRREDPPI) and hormonal therapy initiation by type of analysis and race and ethnicity-stratified subpopulations among HR-positive breast cancer patients in the Pathways Study (n = 3,557) **(removal of stage IV)** | | | | | |
| --- | --- | --- | --- | --- | --- |
| Type of Analysis | SRREDPPI | Hormonal Therapy Initiation | | Univariable Models | Multivariable Models^a^ |
|  |  | No | Yes | OR (95% CI) | aOR (95% CI) |
|  |  | n (col %) | n (col %) |  |  |
| Two-category race and ethnicity variable | Ever | 66 (16.5) | 391 (12.4) | 1.40 (1.04 – 1.85) | 1.44 (1.06 – 1.94) |
|  | Never | 334 (83.5) | 2766 (87.6) |  |  |
| Five-category race and ethnicity variable | Ever | 66 (16.5) | 391 (12.4) | 1.40 (1.04 – 1.85) | 1.51 (1.10 – 2.04) |
|  | Never | 334 (83.5) | 2766 (87.6) |  |  |
| Non-Hispanic White | Ever | 28 (10.1) | 143 (6.7) | 1.57 (1.00 – 2.36) | 1.46 (0.93 – 2.24) |
|  | Never | 249 (89.9) | 1991 (93.3) |  |  |
| Racial or ethnic minority combined | Ever | 38 (30.9) | 248 (24.2) | 1.40 (0.92 – 2.09) | 1.36 (0.88 – 2.06) |
|  | Never | 85 (69.1) | 775 (75.8) |  |  |
| Black | Ever | 10 (47.6) | 63 (33.5) | 1.80 (0.72 – 4.50) | 1.47 (0.55 – 3.90) |
|  | Never | 11 (52.4) | 125 (66.5) |  |  |
| Asian | Ever | 15 (34.9) | 126 (29.5) | 1.28 (0.65 – 2.45) | 1.27 (0.61 – 2.52) |
|  | Never | 28 (65.1) | 301 (70.5) |  |  |
| Hispanic | Ever | 10 (22.2) | 55 (15.8) | 1.53 (0.68 – 3.16) | 1.59 (0.69 – 3.44) |
|  | Never | 35 (77.8) | 294 (84.2) |  |  |
| American Indian, Alaska Native, Pacific Islander | Ever | — | — | 3.75 (0.66 – 19.46) | 20.87 (1.66 – 414.48) |
|  | Never | 11 (78.6) | 55 (93.2) |  |  |
| ^a^Adjusted for covariates including race or ethnicity (primary: NHW vs. WOC; sub-analysis: race or ethnicity strata), country of birth, age, education level, income level, marital status, and AJCC staging.  SRREDPPI = Self-reported racial/ethnic discrimination in patient-provider interactions  OR = odds ratio  aOR = adjusted odds ratio  Two-category race and ethnicity variable = examined the association of non-initiation of hormonal therapy by comparing ever versus never reported discrimination, adjusting for covariates including combined racial or ethnic minority women versus Non-Hispanic White women  Five-category race and ethnicity variable = examined the association of non-initiation of hormonal therapy by comparing ever versus never reported discrimination, adjusted the stratification by each racial/ethnic group of women with Non-Hispanic White women as the reference  To protect patient confidentiality and comply with institutional privacy policies, cell counts ≤5 are not reported and are indicated by '—' in tables. | | | | | |

| Supplemental Table 16. Results of univariable and multivariable logistic regression analyses for ever/never self-reported racial or ethnic discrimination in patient-provider interactions (SRREDPPI) and radiation therapy initiation by type of analysis and race and ethnicity-stratified subpopulations among radiation therapy indicated breast cancer patients in the Pathways Study (n = 3,225) **(removal of stage IV)** | | | | | |
| --- | --- | --- | --- | --- | --- |
| Type of Analysis | SRREDPPI | Radiation Therapy Initiation | | Univariable Models | Multivariable Models^a^ |
|  |  | No | Yes | OR (95% CI) | aOR (95% CI) |
|  |  | n (col %) | n (col %) |  |  |
| Two-category race and ethnicity variable | Ever | 211 (15.2) | 217 (11.8) | 1.35 (1.10 – 1.65) | 1.27 (0.99 – 1.62) |
|  | Never | 1173 (84.8) | 1624 (88.2) |  |  |
| Five-category race and ethnicity variable | Ever | 211 (15.2) | 217 (11.8) | 1.35 (1.10 – 1.65) | 1.22 (0.96 – 1.57) |
|  | Never | 1173 (84.8) | 1624 (88.2) |  |  |
| Non-Hispanic White | Ever | 59 (6.8) | 86 (6.6) | 1.04 (0.73 – 1.46) | 1.17 (0.79 – 1.73) |
|  | Never | 809 (93.2) | 1223 (93.4) |  |  |
| Race or ethnic minority combined | Ever | 152 (29.5) | 131 (24.6) | 1.28 (0.97 – 1.68) | 1.35 (0.98 – 1.86) |
|  | Never | 364 (70.5) | 401 (75.4) |  |  |
| Black | Ever | 45 (35.2) | 46 (38.0) | 0.88 (0.53 – 1.48) | 0.88 (0.48 – 1.60) |
|  | Never | 83 (64.8) | 75 (62.0) |  |  |
| Asian | Ever | 67 (34.7) | 51 (28.3) | 1.35 (0.87 – 2.09) | 1.45 (0.84 – 2.51) |
|  | Never | 126 (65.3) | 129 (71.7) |  |  |
| Hispanic | Ever | 33 (21.0) | 32 (16.7) | 1.33 (0.77 – 2.29) | 1.69 (0.89 – 3.23) |
|  | Never | 124 (79.0) | 160 (83.3) |  |  |
| American Indian, Alaska Native, and Pacific Islander | Ever | 7 (18.4) | — | 4.18 (0.93 – 29.43) | 3.41 (0.41 – 50.94) |
|  | Never | 31 (81.6) | 37 (94.9) |  |  |
| ^a^Adjusted for covariates including race or ethnicity (two-category race and ethnicity variable: Non-Hispanic White vs. racial or ethnic minority women combined; five-category race and ethnicity variable: race or ethnicity strata), country of birth, age at diagnosis, education level, income level, marital status, and American Joint Committee on Cancer staging.  SRREDPPI = self-reported racial/ethnic discrimination in patient-provider interactions  OR = odds ratio  aOR = adjusted odds ratio  Two-category race and ethnicity variable = examined the association of non-initiation of adjuvant therapies by comparing ever versus never reported discrimination, adjusting for combined racial or ethnic minority women versus NHW women  Five-category race and ethnicity variable = examined the association of non-initiation of adjuvant therapies by comparing ever versus never reported discrimination, adjusting the stratification by each racial/ethnic group of women with NHW women as the reference  To protect patient confidentiality and comply with institutional privacy policies, cell counts ≤5 are not reported and are indicated by '—' in tables. | | | | | |

| Supplemental Table 17. Results of univariable and multivariable logistic regression analyses for ever/never self-reported racial or ethnic discrimination in patient-provider interactions (SRREDPPI) and chemotherapy initiation by type of analysis and race and ethnicity-stratified subpopulations among chemotherapy indicated breast cancer patients in the Pathways Study (n = 2413) **(removal of stage IV)** | | | | | |
| --- | --- | --- | --- | --- | --- |
| Type of Analysis | SRREDPPI | Chemotherapy Initiation | | Univariable Models | Multivariable Models^a^ |
|  |  | No | Yes | OR (95% CI) | aOR (95% CI) |
|  |  | n (col %) | n (col %) |  |  |
| Two-category race and ethnicity variable | Ever | 99 (12.7) | 245 (15.0) | 0.82 (0.64 – 1.05) | 0.96 (0.68 – 1.34) |
|  | Never | 683 (87.3) | 1386 (85.0) |  |  |
| Five-category race and ethnicity variable | Ever | 99 (12.7) | 245 (15.0) | 0.82 (0.64 – 1.05) | 0.94 (0.67 – 1.32) |
|  | Never | 683 (87.3) | 1386 (85.0) |  |  |
| Non-Hispanic White | Ever | 35 (6.6) | 55 (5.8) | 1.16 (0.74 – 1.79) | 1.26 (0.72 – 2.19) |
|  | Never | 493 (93.4) | 897 (94.2) |  |  |
| Race or ethnic minority combined | Ever | 64 (25.2) | 190 (28.0) | 0.87 (0.62 – 1.20) | 0.77 (0.50 – 1.19) |
|  | Never | 190 (74.8) | 489 (72.0) |  |  |
| Black | Ever | 21 (35.0) | 49 (34.0) | 1.04 (0.55 – 1.95) | 0.97 (0.44 – 2.15) |
|  | Never | 39 (65.0) | 95 (66.0) |  |  |
| Asian | Ever | 24 (23.3) | 96 (35.0) | 0.56 (0.33 – 0.94) | 0.56 (0.28 – 1.09) |
|  | Never | 79 (76.7) | 178 (65.0) |  |  |
| Hispanic | Ever | 18 (23.1) | 38 (17.8) | 1.38 (0.72 – 2.57) | 0.94 (0.34 – 2.61) |
|  | Never | 60 (76.9) | 175 (82.2) |  |  |
| American Indian, Alaska Native, Pacific Islander | Ever | — | 7 (14.6) | 0.49 (0.02 – 3.15) | 2.95 (0.04 – 143.77) |
|  | Never | 12 (92.3) | 41 (85.4) |  |  |
| ^a^Adjusted for covariates including race or ethnicity (two-category race and ethnicity variable: Non-Hispanic White vs. racial or ethnic minority women combined; five-category race and ethnicity variable: race or ethnicity strata), country of birth, age, education level, income level, marital status, American Joint Committee on Cancer staging, hormone receptor (HR) status, and human epidermal growth factor receptor 2 (HER2) status.  SRREDPPI = Self-reported racial/ethnic discrimination in patient-provider interactions  OR = odds ratio  aOR = adjusted odds ratio  Two-category race and ethnicity variable = examined the association of non-initiation of adjuvant therapies by comparing ever versus never reported discrimination, adjusting for combined racial or ethnic minority women versus Non-Hispanic White women  Five-category race and ethnicity variable = examined the association of non-initiation of adjuvant therapies by comparing ever versus never reported discrimination, adjusting the stratification by each racial/ethnic group of women with Non-Hispanic White women as the reference  To protect patient confidentiality and comply with institutional privacy policies, cell counts ≤5 are not reported and are indicated by '—' in tables. | | | | | |

| Supplemental Table 18. Results of multivariable logistic regression analyses (with and without adjusting for Charlson Comorbidity Index) for ever/never self-reports of racial or ethnic discrimination in patient-provider interactions (SRREDPPI) and hormonal therapy initiation by type of analysis and race and ethnicity-stratified subpopulations among HR-positive breast cancer patients in the Pathways Study (n = 3610) | | | | | |
| --- | --- | --- | --- | --- | --- |
| Type of Analysis | SRREDPPI | Hormonal therapy Initiation | | Multivariable Models^a^ | Multivariable Models^b^ |
|  |  | No | Yes | OR (95% CI) | aOR (95% CI) |
|  |  | n (col %) | n (col %) |  |  |
| Two-category race and ethnicity variable | Ever | 66 (16.3) | 395 (12.3) | 1.43 (1.05 – 1.93) | 1.49 (1.05 – 1.92) |
|  | Never | 338 (83.7) | 2811 (87.7) |  |  |
| Five-category race and ethnicity variable | Ever | 66 (16.3) | 395 (12.3) | 1.50 (1.10 – 2.02) | 1.49 (1.09 – 2.02) |
|  | Never | 338 (83.7) | 2811 (87.7) |  |  |
| Non-Hispanic White | Ever | 28 (10.0) | 145 (6.7) | 1.44 (0.91 – 2.21) | 1.46 (0.92 – 2.23) |
|  | Never | 252 (90.0) | 2019 (93.3) |  |  |
| Racial or ethnic minority combined | Ever | 38 (30.6) | 250 (24.0) | 1.35 (0.88 – 2.05) | 1.35 (0.87 – 2.04) |
|  | Never | 86 (69.4) | 792 (76.0) |  |  |
| Black | Ever | 10 (47.6) | 64 (33.0) | 1.47 (0.55 – 3.90) | 1.48 (0.55 – 3.97) |
|  | Never | 11 (52.4) | 130 (67.0) |  |  |
| Asian | Ever | 15 (34.9) | 126 (29.2) | 1.27 (0.61 – 2.52) | 1.29 (0.62 – 2.58) |
|  | Never | 28 (65.1) | 306 (70.8) |  |  |
| Hispanic | Ever | 10 (21.7) | 55 (15.4) | 1.63 (0.70 – 3.51) | 1.60 (0.68 – 3.50) |
|  | Never | 36 (78.3) | 301 (84.6) |  |  |
| American Indian, Alaska Native, Pacific Islander | Ever | — | — | 20.87 (1.66 – 414.47) | 26.35 (1.94 – 633.78) |
|  | Never | 11 (78.6) | 55 (91.7) |  |  |
| ^a^Adjusted for covariates including race or ethnicity (primary: NHW vs. WOC; sub-analysis: race or ethnicity strata), country of birth, age, education level, income level, marital status, and AJCC staging.  ^b^Adjusted for covariates including race or ethnicity (primary: NHW vs. WOC; sub-analysis: race or ethnicity strata), country of birth, age, education level, income level, marital status, AJCC staging, and Charlson Comorbidity Index (12 months before and 3 months after the date of diagnosis).  SRREDPPI = Self-reported racial/ethnic discrimination in patient-provider interactions  OR = odds ratio  aOR = adjusted odds ratio  col = column  Two-category race and ethnicity variable = examined the association of non-initiation of hormonal therapy by comparing ever versus never reported discrimination, adjusting for covariates including combined racial or ethnic minority women versus Non-Hispanic White women  Five-category race and ethnicity variable = examined the association of non-initiation of hormonal therapy by comparing ever versus never reported discrimination, adjusted the stratification by each racial/ethnic group of women with Non-Hispanic White women as the reference.  To protect patient confidentiality and comply with institutional privacy policies, cell counts ≤5 are not reported and are indicated by '—' in tables. | | | | | |

| Supplemental Table 19. Results of multivariable logistic regression analyses (with and without adjusting for Charlson Comorbidity Index) for ever/never self-reported racial or ethnic discrimination in patient-provider interactions (SRREDPPI) and chemotherapy initiation by type of analysis and race and ethnicity-stratified subpopulations among chemotherapy indicated breast cancer patients in the Pathways Study (n = 2450) | | | | | |
| --- | --- | --- | --- | --- | --- |
| Type of Analysis | SRREDPPI | Chemotherapy Initiation | | Multivariable Models^a^ | Multivariable Models^b^ |
|  |  | No | Yes | OR (95% CI) | aOR (95% CI) |
|  |  | n (col %) | n (col %) |  |  |
| Two-category race and ethnicity variable | Ever | 99 (12.6) | 247 (14.9) | 0.96 (0.69 – 1.33) | 0.95 (0.67 – 1.32) |
|  | Never | 689 (87.4) | 1415 (85.1) |  |  |
| Five-category race and ethnicity variable | Ever | 99 (12.6) | 247 (14.9) | 0.94 (0.67 – 1.32) | 0.93 (0.66 – 1.31) |
|  | Never | 689 (87.4) | 1415 (85.1) |  |  |
| Non-Hispanic White | Ever | 35 (6.6) | 56 (5.8) | 1.24 (0.71 – 2.16) | 1.23 (0.70 – 2.12) |
|  | Never | 496 (93.4) | 911 (94.2) |  |  |
| Race or ethnic minority combined | Ever | 64 (24.9) | 191 (27.5) | 0.79 (0.51 – 1.20) | 0.78 (0.51 – 1.20) |
|  | Never | 193 (75.1) | 504 (72.5) |  |  |
| Black | Ever | 21 (35.0) | 50 (32.9) | 0.98 (0.44 – 2.15) | 1.01 (0.45 – 2.25) |
|  | Never | 39 (65.0) | 102 (67.1) |  |  |
| Asian | Ever | 24 (23.3) | 96 (34.5) | 0.56 (0.28 – 1.09) | 0.56 (0.28 – 1.09) |
|  | Never | 79 (76.7) | 182 (65.5) |  |  |
| Hispanic | Ever | 18 (22.2) | 38 (17.5) | 1.05 (0.39 – 2.81) | 1.05 (0.39 – 2.85) |
|  | Never | 63 (77.8) | 179 (82.5) |  |  |
| American Indian, Alaska Native, Pacific Islander | Ever | — | 7 (14.6) | 2.95 (0.04 – 143.77) | NA |
|  | Never | 12 (92.3) | 41 (85.4) |  |  |
| ^a^Adjusted for covariates including race or ethnicity (two-category race and ethnicity variable: Non-Hispanic White vs. racial or ethnic minority women combined; five-category race and ethnicity variable: race or ethnicity strata), country of birth, age, education level, income level, marital status, American Joint Committee on Cancer staging, hormone receptor (HR) status, and human epidermal growth factor receptor 2 (HER2) status.  ^b^Adjusted for covariates including race or ethnicity (two-category race and ethnicity variable: Non-Hispanic White vs. racial or ethnic minority women combined; five-category race and ethnicity variable: race or ethnicity strata), country of birth, age, education level, income level, marital status, American Joint Committee on Cancer staging, hormone receptor (HR) status, human epidermal growth factor receptor 2 (HER2) status, and Charlson Comorbidity Index (12 months before and 3 months after the date of diagnosis).  SRREDPPI = Self-reported racial/ethnic discrimination in patient-provider interactions  OR = odds ratio  aOR = adjusted odds ratio  col = column  Two-category race and ethnicity variable = examined the association of non-initiation of adjuvant therapies by comparing ever versus never reported discrimination, adjusting for combined racial or ethnic minority women versus Non-Hispanic White women  Five-category race and ethnicity variable = examined the association of non-initiation of adjuvant therapies by comparing ever versus never reported discrimination, adjusting the stratification by each racial/ethnic group of women with Non-Hispanic White women as the reference.  To protect patient confidentiality and comply with institutional privacy policies, cell counts ≤5 are not reported and are indicated by '—' in tables. | | | | | |

Supplemental Figure 1. Inclusion criteria for the analysis of self-reported racial/ethnic discrimination in patient-provider interactions in the Pathways Study, for participants with responses to the two questions at baseline, 6-month, and 24-month follow-ups and clinically indicated adjuvant therapies (final sample for hormonal therapy n = 3610; chemotherapy n = 2450; radiation therapy = 3258)
